# iRhom2 is essential for innate immunity to RNA virus by antagonizing ER- and mitochondria-associated degradation of VISA

**DOI:** 10.1371/journal.ppat.1006693

**Published:** 2017-11-20

**Authors:** Wei-Wei Luo, Shu Li, Chen Li, Zhou-Qin Zheng, Pan Cao, Zhen Tong, Huan Lian, Su-Yun Wang, Hong-Bing Shu, Yan-Yi Wang

**Affiliations:** 1 Key Laboratory of Special Pathogens and Biosafety, Wuhan Institute of Virology, Chinese Academy of Sciences, Wuhan, China; 2 Medical Research Institute, State Key Laboratory of Virology, School of Medicine, Wuhan University, Wuhan, China; 3 College of Life Sciences, Wuhan University, Wuhan, China; University of Southern California, UNITED STATES

## Abstract

VISA (also known as MAVS, IPS-1 and Cardif) is an essential adaptor protein in innate immune response to RNA virus. The protein level of VISA is delicately regulated before and after viral infection to ensure the optimal activation and timely termination of innate antiviral response. It has been reported that several E3 ubiquitin ligases can mediate the degradation of VISA, but how the stability of VISA is maintained before and after viral infection remains enigmatic. In this study, we found that the ER-associated inactive rhomboid protein 2 (iRhom2) plays an essential role in mounting an efficient innate immune response to RNA virus by maintaining the stability of VISA through distinct mechanisms. In un-infected and early infected cells, iRhom2 mediates auto-ubiquitination and degradation of the E3 ubiquitin ligase RNF5 and impairs the assembly of VISA-RNF5-GP78 complexes, thereby antagonizes ER-associated degradation (ERAD) of VISA. In the late phase of viral infection, iRhom2 mediates proteasome-dependent degradation of the E3 ubiquitin ligase MARCH5 and impairs mitochondria-associated degradation (MAD) of VISA. Maintenance of VISA stability by iRhom2 ensures efficient innate antiviral response at the early phase of viral infection and ready for next round of response. Our findings suggest that iRhom2 acts as a checkpoint for the ERAD/MAD of VISA, which ensures proper innate immune response to RNA virus.

## Author summary

VISA is a central adaptor in innate immune response to RNA virus, which is down-regulated by multiple ubiquitination-dependent mechanisms. In this study, we found that the ER-associated protein iRhom2 promotes VISA stability by suppressing ER- and mitochondria-associated degradation pathways in early- and late-infected cells respectively, thereby plays an essential role in efficient innate immune response to RNA virus.

## Introduction

Innate immune response provides the first line of host defense against invading microbial pathogens[1]. Sensing of pathogen-derived nucleic acids via pattern-recognition receptors (PRRs) is a general strategy used by host cells to detect invading pathogens, which initiates a series of cellular signaling events that ultimately induce the expression of type I interferons (IFNs), proinflammatory cytokines and other anti-microbial effector proteins [1,2].

RNA viruses are a class of major pathogens that cause severe infectious and immunological diseases as well as death. Upon infection, viral RNAs are detected by cytoplasmic sensors including RIG-I and MDA5, which are RNA helicase proteins that exhibit different ligand specificities. Binding of viral RNAs to RIG-I and MDA5 induces their conformational changes and recruitment to the mitochondrial membrane-located adaptor protein VISA (also called MAVS, IPS-1 and Cardif)[3–6]. This triggers the formation of large prion-like VISA polymers, which in turn serve as platforms for recruitment of TRAF2/3/5/6 through its TRAF-binding motifs[7,8]. The TRAF proteins further recruit TBK1 and the IKK complex to phosphorylate IRF3 and IκBα respectively, leading to activation of IRF3 and NF-κB and induction of downstream antiviral effectors.

Although VISA-mediated signaling is required for efficient innate immune responses to RNA viruses, it must be tightly regulated to prevent excessive response that causes tissue damage and death [9,10]. It has been demonstrated that several proteins, including the NLR family member X1 (NLRX1), UBXN1, and G patch domain-containing protein 3 (GPATCH3), negatively regulate VISA-mediated antiviral responses by impairing the assembly of VISA-associated complexes [11–13]. In addition, several E3 ubiquitin ligases, including RNF5, AIP4/ITCH, MARCH5, and Smurf1, attenuate innate immune responses to RNA viruses by promoting K48-linked polyubiquitination and proteasomal degradation of VISA [14–17]. How the stability of VISA is maintained before and after viral infection remains enigmatic. In addition, it has been well established that VISA is primarily localized at the contact sites of mitochondria and the ER, which is called mitochondrion-associated membrane (MAM) [18]. The significance of the MAM localization of VISA and roles of the ER on regulation of VISA-mediated signaling remain enigmatic.

ER-associated degradation (ERAD) is an evolutionarily conserved protein quality-control mechanism that eliminates misfolded proteins from the ER in eukaryotic cells [19]. Mitochondrion-associated degradation (MAD) is analogous to the ERAD pathway in that they both require the valosin-containing protein (VCP)/p97 to dislodge ubiquitinated proteins from organelle membranes and escort their degradation by the proteasomes [20]. In this study, we found that the ERAD/MAD dynamically regulated the protein level of VISA before and after viral infection, which was subverted by the ER-associated inactive rhomboid protein 2 (iRhom2). In un-infected and early infected cells, iRhom2 mediates auto-ubiquitination and degradation of RNF5 and impairs ERAD of VISA. In the late phase of viral infection, iRhom2 mediates proteasome-dependent degradation of MARCH5 and impairs MAD of VISA. Our findings suggest that iRhom2 acts as a checkpoint for the ERAD/MAD of VISA, which ensures proper innate immune response to RNA virus.

## Results

### iRhom2 positively regulates RNA virus-triggered signaling

**Fig 1:**
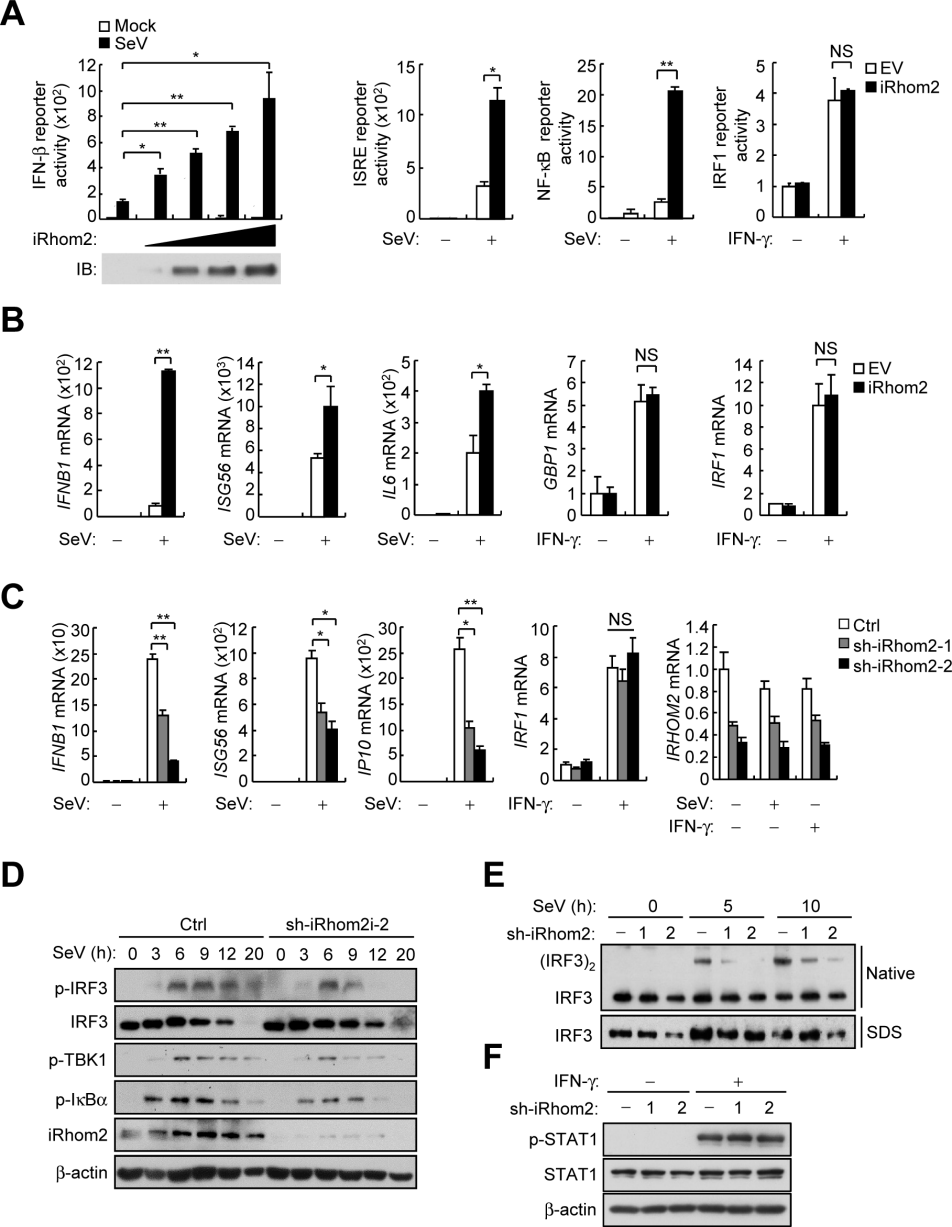
Identification of iRhom2 as a positive regulator of SeV virus-triggered signaling. (A) Luciferase reporter assays with HEK293 cells that were co-transfected with the indicated reporter plasmids plus iRhom2 or empty vector (EV) and then left untreated (Mock) or treated with SeV or IFN-γ (100 ng/ml) for 12 h. (B) qPCR analysis of *IFNB1*, *ISG56*, *IL6*, *GBP1* and *IRF1* mRNAs in HEK293 cells that were transfected with iRhom2 or empty vector (EV) and then treated with SeV for 8 h or IFN-γ (100 ng/ml) for 6 h. (C) qPCR analysis of *IFNB1*, *ISG56*, *IP10*, *IRF1* and *IRHOM2* mRNAs in HEK293 cells that were transfected with control shRNA (Ctrl) and iRhom2-shRNA plasmid and then selected with puromycin (1 μg/ml) for 2 days before treatment with SeV for 8 h or IFN-γ (100 ng/ml) for 6 h. (D) Immunoblot analysis of the indicated proteins with HEK293 cells that were transfected with iRhom2-shRNA plasmid and selected with puromycin (1 μg/ml) for 2 days and then infected with SeV for the indicated times. (E) Native gel analysis (above) and SDS-PAGE (below) of IRF3 dimerization with HEK293T cells that were transfected with iRhom2-shRNA plasmid and selected with puromycin (1 μg/ml) for 2 days and then infected with SeV for the indicated times. (F)Immunoblot analysis of the indicated proteins with HEK293T cells that were transfected with iRhom2-shRNA plasmid and selected with puromycin (1 μg/ml) for 2 days and then treated with IFN-γ (100 ng/ml) for the indicated times. *p < 0.05; **p < 0.01 (unpaired *t*-test). Data are representative of three experiments with similar results.

Recently, we have demonstrated that the ER-associated iRhom2 plays an essential role in innate immune response to DNA virus by promoting the stability and cellular trafficking of MITA/STING, a central adaptor in DNA-triggered induction of downstream antiviral genes [21]. To investigate whether iRhom2 plays a role in innate immune responses to RNA viruses, we firstly performed reporter assays. The results showed that overexpression of iRhom2 facilitated Sendai virus (SeV)-triggered activation of the *IFNB* promoter, ISRE and NF-κB, but not IFN-γ-induced activation of the *IRF1* promoter (**Fig 1A**). Quantitative real-time PCR (qPCR) analysis indicated that overexpression of iRhom2 increased the mRNA levels of *IFNB1*, *ISG56* and *IL6* genes induced by SeV infection, but not the mRNA levels of *GBP1* or *IRF1* genes induced by IFNγ (**Fig 1B**). Conversely, knockdown of iRhom2 inhibited SeV-induced transcription of *IFNB1*, *ISG56* and *IP10* genes, but not IFN-γ-induced transcription of *IRF1* gene (**Fig 1C**). Consistently, knockdown of iRhom2 decreased SeV-induced phosphorylation of IRF3, TBK1 and IκBα (**Fig 1D**), and dimerization of IRF3 (**Fig 1E**), but had no marked effects on IFN-γ-induced phosphorylation of STAT1 (**Fig 1F**). These results suggest that iRhom2 positively regulates RNA virus-triggered induction of antiviral genes.

**Fig 2:**
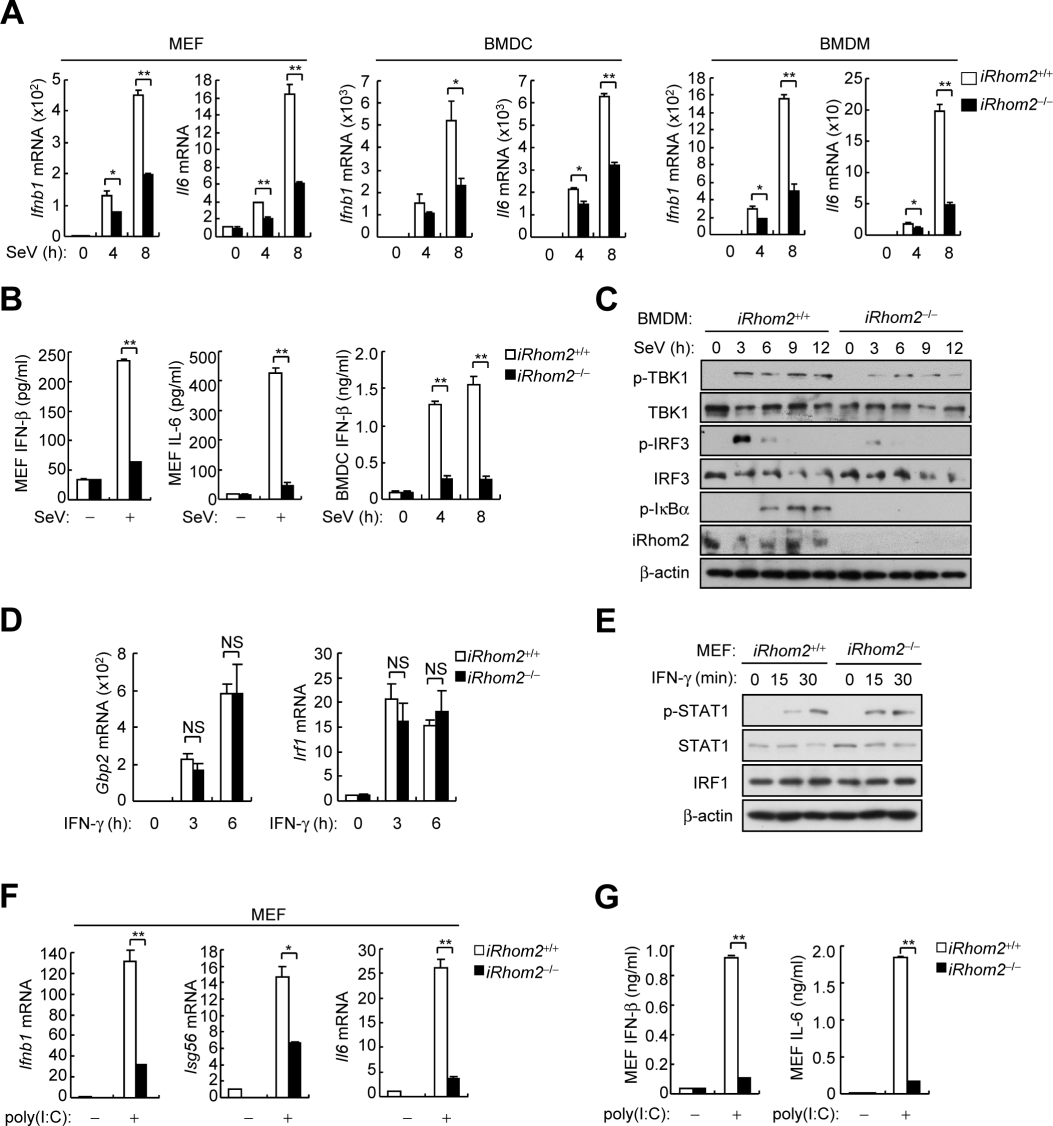
iRhom2 is essential for SeV-triggered induction of downstream antiviral genes. (A) qPCR analysis of *Ifnb1* and *Il6* mRNAs in *iRhom2*^*+/+*^ and *iRhom2*^*‒/‒*^ MEFs (left), BMDCs (middle) and BMDMs (right) infected with SeV for the indicated times. (B) ELISA of secreted IFN-β and IL-6 in *iRhom2*^+/+^ or *iRhom2*^*‒/‒*^ cells un-infected or infected with SeV for 8 h (MEF) or the indicated times (BMDCs). (C) Immunoblot analysis of the indicated proteins in *iRhom2*^+/+^ and *iRhom2*^–/–^BMDMs un-infected or infected with SeV for the indicated times. (D) qPCR analysis of *Gbp1* and *Irf1* mRNAs in *iRhom2*^+/+^ or *iRhom2*^*‒/‒*^ MEFs untreated or treated with IFN-γ (100 ng/ml) for the indicated times. (E) Immunoblot analysis of the indicated proteins in *iRhom2*^+/+^ and *iRhom2*^–/–^MEFs untreated or treated with IFN-γ (100 ng/ml) for the indicated times. (F) qPCR analysis of *Ifnb1*, *Isg56* and *Il6* mRNAs in *iRhom2*^+/+^ and *iRhom2*^–/–^MEFs transfected with poly (I:C) (3 μg/ml) for 6 h. (G) ELISA of secreted IFN-β and IL-6 in *iRhom2*^+/+^ or *iRhom2*^*‒/‒*^ MEFs transfected with poly (I:C) (3 μg/ml) for 6 h. *P < 0.05; **P < 0.01 (unpaired *t*-test). Data are representative of three experiments with similar results.

To investigate whether iRhom2 is required for innate immune responses to RNA viruses, we determined the effects of iRhom2-deficiency on induction of downstream antiviral genes in *iRhom2*^*‒/‒*^ murine embryonic fibroblasts (MEFs), lung fibroblasts (MLFs), bone-marrow-derived dendrite cells (BMDCs) and bone-marrow-derived macrophages (BMDMs). We found that induction of *Ifnb1* and *Il6* mRNAs by SeV was severely impaired in *iRhom2*^*‒/‒*^MEFs, MLFs, BMDCs and BMDMs in comparison with their wild-type counterparts (**Fig 2A and S1A Fig**). In similar experiments, the mRNA levels of *Ifnb1*, *Isg56* and *Il6* genes induced by other RNA viruses including vesicular stomatitis virus (VSV) and Newcastle disease virus (NDV) were also markedly inhibited in *iRhom2*^*‒/‒*^MEFs and BMDCs (**S1B and S1C Fig**). Consistently, secretion of IFN-β and IL-6 was remarkably inhibited in *iRhom2*^*‒/‒*^MEFs and BMDCs after SeV infection (**Fig 2B)**. The phosphorylation of TBK1, IRF3 and IκBα induced by SeV was also markedly inhibited in *iRhom2*^*‒/‒*^BMDMs (**Fig 2C)**. However, IFN-γ-induced expression of *Gbp1* and *Irf1* mRNAs (**Fig 2D**) and accumulation of phosphorylated STAT1 (**Fig 2E**) were comparable between *iRhom2*^*‒/‒*^and *iRhom2*^*+/+*^cells. In addition to viral infection, we also examined the effects of iRhom2-deficiency on expression of antiviral genes induced by transfected synthetic RNA analog poly(I:C). The results indicated that iRhom2-deficiency also markedly inhibited the levels of *Ifnb1*, *Isg56* and *Il6* mRNAs (**Fig 2F**) as well secreted IFN-β and IL-6 (**Fig 2G**) induced by cytoplasmic poly(I:C). Collectively, these data suggest that iRhom2 is required for efficient induction of antiviral genes by RNA viruses and cytoplasmic poly(I:C) in murine fibroblasts and immune cells.

### iRhom2 is essential for innate immune responses to RNA viruses *in vivo*

**Fig 3:**
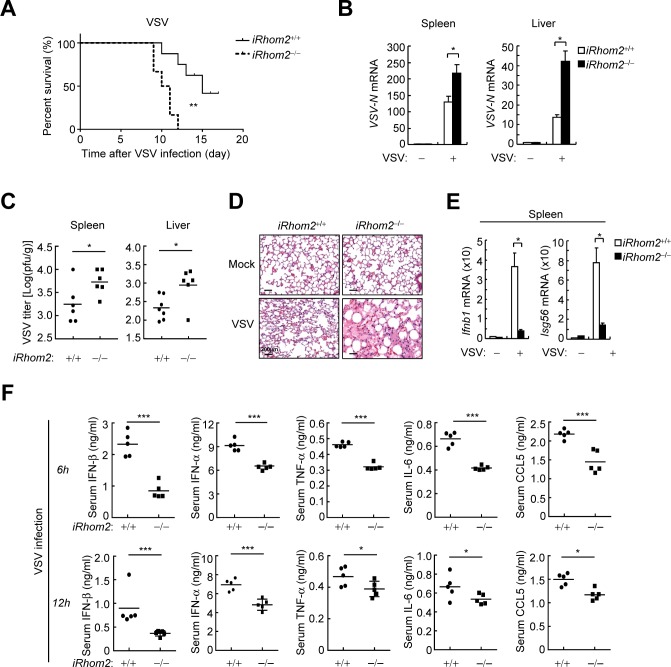
iRhom2 is essential for host defense against VSV infection in mice. (A)Survival of *iRhom2*^*‒/‒*^ and *iRhom2*^–/–^mice (*n* = 7 per strain, 8 weeks old) injected intraperitoneally with VSV (1×10^8^ PFU per mouse). (**B**) qPCR analysis of VSV RNA in the spleens and livers of *iRhom2*^*‒/‒*^ and *iRhom2*^–/–^mice (*n* = 3 per strain, 8 weeks old) injected intraperitoneally with VSV (1×10^8^ PFU per mouse) 12 h before. (**C**) Measurement of VSV titer in the spleens and livers of *iRhom2*^*‒/‒*^ and *iRhom2*^–/–^mice (*n* = 6 per strain, 8 weeks old) injectied intraperitoneally with VSV (5×10^8^ PFU per mouse) 24 h before by plaque assay. **(D)** qPCR analysis of *Ifnb1* and *Isg56* mRNAs in the spleens of *iRhom2*^*‒/‒*^ and *iRhom2*^–/–^mice (*n* = 3 per strain, 8 weeks old) injected intraperitoneally with VSV (1×10^8^ PFU per mouse). **(E)** Hematoxylin-and-eosin-stained lung sections from *iRhom2*^*‒/‒*^ and *iRhom2*^–/–^mice (*n* = 3 per strain, 8 weeks old) injected intraperitoneally with VSV (1×10^8^ PFU per mouse). Scale bars, 200 μm. **(F)** ELISA of the indicated cytokines in the sera of *iRhom2*^*‒/‒*^ and *iRhom2*^–/–^mice (*n* = 5 per strain, 8 weeks old) injected intravenously with HSV-1 (1×10^7^ PFU per mouse) for 6 and 12 h. *P < 0.05; **P < 0.01 (unpaired *t*-test). Data are representative of three experiments with similar results.

To evaluate the importance of iRhom2 in host defense to RNA viruses *in vivo*, we infected wild-type and iRhom2-defcient mice with VSV. All infected *iRhom2*^*‒/‒*^ mice developed discrepant lethargy and ataxia within 8 days of VSV infection and died within 4 days of the appearance of symptoms (**Fig 3A)**. In these experiments, only approximately 60% of infected wild-type mice exhibited the symptoms, which then died over a period of 9–17 days after the appearance of symptoms (**Fig 3A)**. Consistently, VSV titers in the liver and spleens from *iRhom2*^*‒/‒*^mice were significantly increased compared to those from *iRhom2*^*‒/‒*^mice (**Fig 3B and 3C)**. Hematoxylin-and-eosin staining showed greater infiltration of immune cells and damage in the lungs of *iRhom2*^*‒/‒*^ in comparison to *iRhom2*^*‒/‒*^mice after infection with VSV (**Fig 3D)**. Moreover, the levels of *Ifnb1* and *Isg56* mRNAs induced by VSV were markedly down-regulated in the spleens of *iRhom2*^*‒/‒*^mice in comparison to those of wild-type mice (**Fig 3E)**. The sera from *iRhom2*^*‒/‒*^mice infected with VSV had significantly lower levels of IFN-α, IFN-β, TNF-α, IL-6 and CCL5 compared with these from wild-type mice (**Fig 3F)**. These results suggest that iRhom2 is essential for host defense against RNA virus infection *in vivo*.

### iRhom2 interacts with VISA

**Fig 4:**
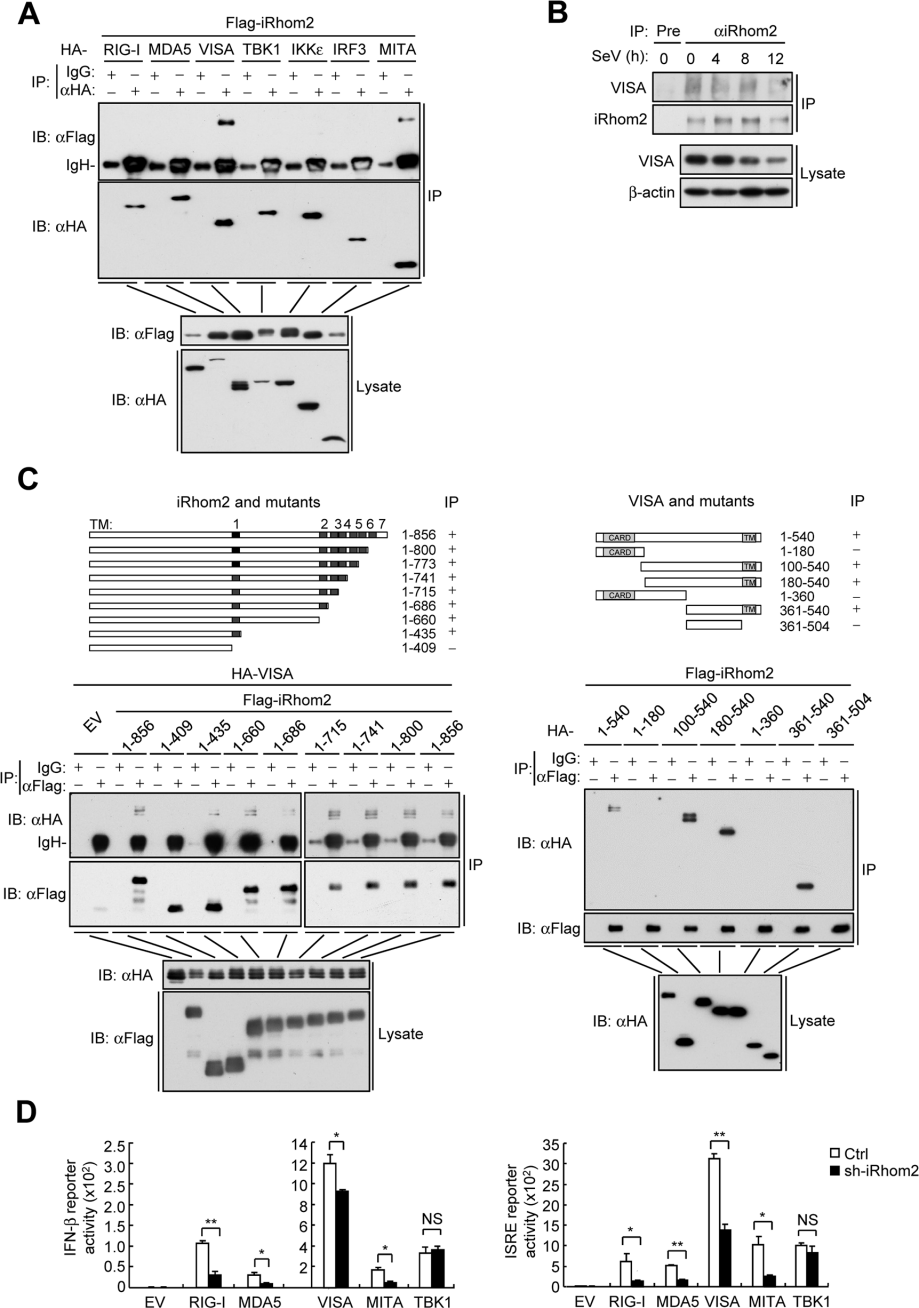
iRhom2 interacts with VISA. (A) Co-immunoprecipitation and immunoblot analysis of HEK293 cells transfected with the indicated plasmids with the indicated antibodies. (B) Detection of the association betweem iRhom2 and VISA by endogenous co-immunoprecipitation and immunoblot analysis with the indicated antibodies in HEK293 cells that were transfected with iRhom2 plasmids and then left un-infected or infected with SeV for the indicated times. (C) Domain mapping of iRhom2-VISA interaction. HEK293 cells were transfected with the indicated truncations before co-immunoprecipitation and immunoblot analysis with the indicated antibodies. The schematic representations of iRhom2 and VISA truncations were shown at the top. (D) Reporter assays for *IFNB* promoter activity in HEK293 cells that were transfected with iRhom2-shRNA plasmid for 20 h, selected with puromycin (1 μg/ml) and then re-transfected with the IFN-β reporter and indicated plasmids for 24 h. *P < 0.05; **P < 0.01 (unpaired *t*-test). Data are representative of three experiments with similar results.

We next investigated the molecular mechanisms responsible for the roles of iRhom2 in innate immune responses to RNA viruses. In transient transfection and co-immunoprecipitation experiments, iRhom2 was associated with VISA and MITA/STING, but not with RIG-I, MDA5, TBK1, IKKε or IRF3 (**Fig 4A)**. Furthermore, results of endogenous co-immunoprecipitation experiments showed that iRhom2 was constitutively associated with VISA before and after viral infection (**Fig 4B**). Confocal microscopy experiments indicated that iRhom2 partially colocalized with VISA in un-infected and SeV-infected cells (**S2A Fig**). In addition, cell fractionation analysis also showed that iRhom2 and VISA were both detected in the mitochondria and ER-containing membrane fractions before and after SeV infection (**S2B Fig**). It has been reported that VISA partially localized in mitochondrial-associated ER membranes (MAMs) [18]. These results suggest that iRhom2 colocalized and interacted with VISA at mitochondria and MAMs.

Domain mapping experiments demonstrated that aa410-430 (the first transmembrane domain) of iRhom2 and the transmembrane domain of VISA are required for their association (**Fig 4C).** Interestingly, the first transmembrane domain of iRhom2 was also responsible for its interaction with MITA [21]. Since the transmembrane domains of VISA, MITA and iRhom2 all form helical structures, it is possible that the interaction between the transmembrane domain of iRhom2 and that of VISA and MITA occurs through helix-helix interactions [22]. Results of reporter assays showed that knockdown of iRhom2 inhibited RIG-I-, MDA5-, VISA-, and MITA- but not TBK1-mediated activation of the *IFNB* promoter and ISRE (**Fig 4D)**, while truncations of iRhom2 that could interact with VISA potentiated SeV-induced activation of the *IFNB* promoter or transcription of *IFNB* gene **(S3 Fig**). These results suggest that iRhom2 acts at the level of VISA in innate immune responses to RNA viruses.

### iRhom2 antagonizes RNF5-mediated degradation of VISA in un-infected and early-infected cells

**Fig 5:**
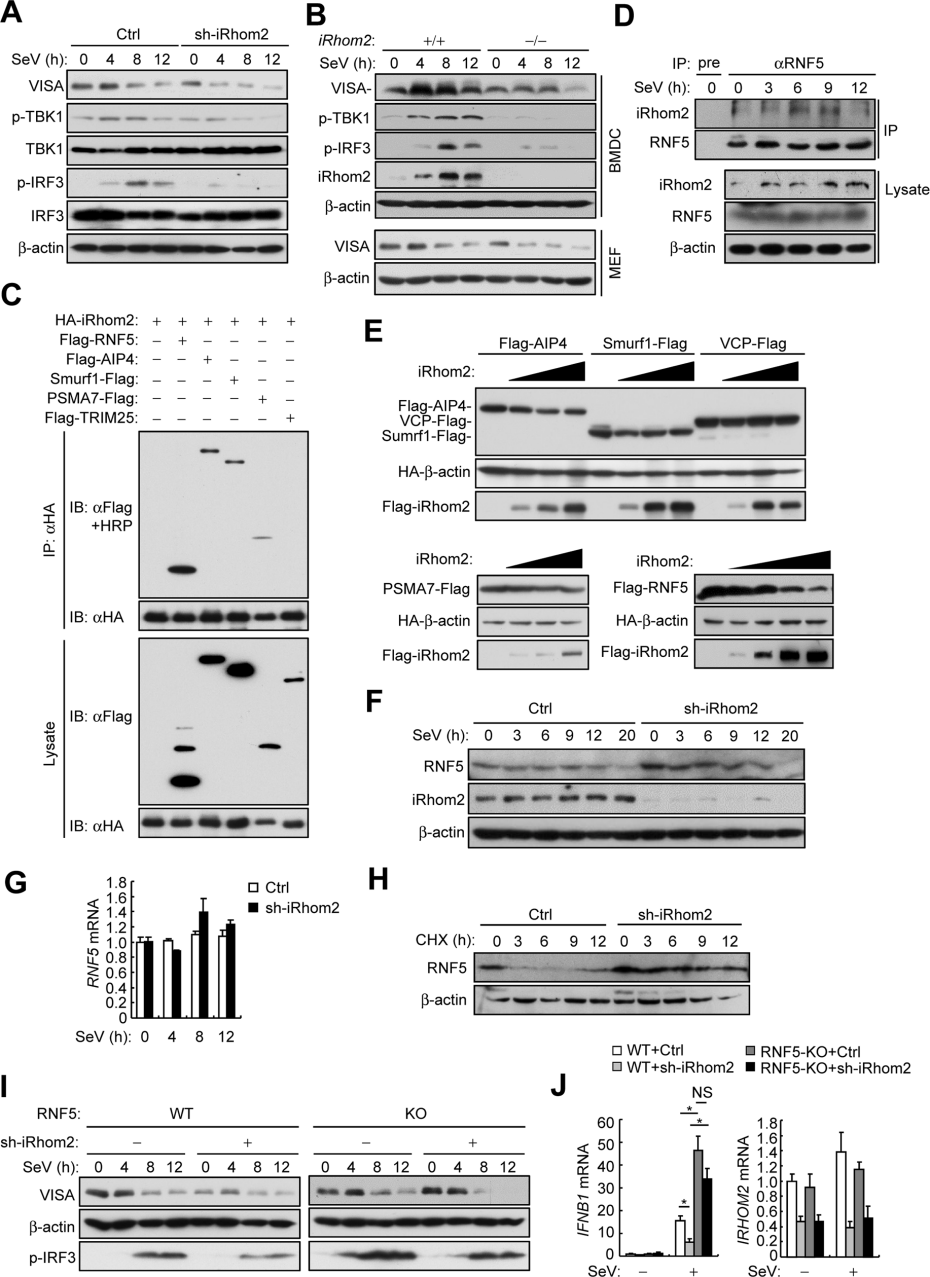
iRhom2 antagonizes RNF5-mediated degradation of VISA. (**A**) Immunoblot analysis of the indicated proteins in HEK293 cells that were transfected with iRhom2-shRNA plasmid and selected with puromycin (1 μg/ml) for 2 days before infected with SeV for the indicated times. (B) Immunoblot analysis of the indicated proteins in *iRhom2*^+/+^ and *iRhom2*^–/–^BMDCs infected with SeV for the indicated times. (C) Detection of the association of iRhom2 with several E3 ligases in HEK293T cells transfected with the indicated plasmids by co-immunoprecipitation and immunoblot analysis with the indicated antibodies. (D) Detection of the association of iRhom2 with RNF5 by endogenous co-immunoprecipitation and immunoblot analysis with the indicated antibodies in HEK293 cells un-infected or infected with HSV-1 for the indicated times. (E) Detection of effects of iRhom2 on the expression of AIP4, Sumrf1, VCP, PSMA7 and RNF5 in HEK293T transfected with the indicated plasmids by co-immunoprecipitation and immunoblot analysis were performed with the indicated antibodies. (F) Immunoblot analysis of the indicated proteins in HEK293T cells that were transfected with iRhom2-shRNA plasmid and selected with puromycin (1 μg/ml) for 2 days before infected with SeV for the indicated times. (G) qPCR analysis of *RNF5* mRNA in HEK293T cells that were transfected with iRhom2-RNAi plasmid and selected with puromycin (1 μg/ml) for 36 h before infected with SeV for the indicated times. (H) Immunoblot analysis of the indicated proteins in HEK293 cells that were transfected with iRhom2-shRNA plasmid and selected with puromycin (1 μg/ml) for 2 days before treated with cycloheximide (CHX; 100 μg/ml) for the indicated times. (I) Immunoblot analysis of the indicated proteins in wild-type and RNF5-KO HEK293T cells transfected with iRhom2-shRNA plasmid for 2 days before infected with SeV for the indicated times. (J) qPCR analysis of *IFNB1* and *IRHOM2* mRNAs in wild-type and RNF5-KO HEK293T cells transfected with iRhom2-shRNA plasmid for 2 days before infected with SeV for 5 h. *P < 0.05; **P < 0.01 (unpaired *t*-test). Data are representative of three experiments with similar results.

Previously, we found that iRhom2 maintains the stability of MITA/STING in viral DNA-triggered signaling pathways [21]. We therefore determined whether iRhom2 regulates the level of VISA in viral RNA-triggered signaling. We found that knockdown of iRhom2 by shRNA in HEK 293 cells (**Fig 5A**) or complete knockout of iRhom2 in BMDCs and MEFs (**Fig 5B**) resulted in lower levels of VISA in un-infected as well as infected cells. Correspondingly, the SeV-induced phosphorylation of TBK1 and IRF3 but not their protein levels were also impaired in iRhom2 knockdown or knockout cells in comparison to the control cells (**Fig 5A and 5B**). These results suggest that iRhom2 plays a role in maintaining the stability of VISA.

Several E3 ligases, including AIP4, RNF5, TRIM25, Smurf1/2, and PSMA7, have been identified to mediate VISA degradation by catalyzing its K48-linked ployubiquitination [14–16,23,24]. This prompted us to test whether iRhom2 promotes the stability of VISA by antagonizing the functions of one or more of these E3 ubiquitin ligases. In co-immunoprecipitation experiments, we found that iRhom2 was associated with RNF5, AIP4, Smurf1 and PSMA7 but not TRIM25 (**Fig 5C)**. Interestingly, we observed that iRhom2 caused shift of RNF5 but not other examined E3 ligases to higher molecular species (**Fig 5C)**. Results of endogenous co-immunoprecipitation experiment also confirmed the interaction between iRhom2 and RNF5. Interestingly, such interaction was impaired at the late phase of infection (**Fig 5D)**. Subsequent co-transfection experiments indicated that overexpression of iRhom2 specifically decreased the level of RNF5 but not other examined E3 ligases in a dose-dependent manner (**Fig 5E)**. Conversely, knockdown of iRhom2 increased the protein level of RNF5 in un-infected or early-infected (3–9 h) cells (**Fig 5F**), but had no marked effects on the mRNA level of *RNF5* (**Fig 5G)**. Inhibition of protein synthesis by cycloheximide indicated that knockdown of iRhom2 promoted the stability of endogenous RNF5 in un-infected cells (**Fig 5H)**. These results suggest that iRhom2 down-regulates RNF5 at the post-translational level in un-infected and early-infected cells.

To determine whether iRhom2 maintaining the stability of VISA by down-regulating RNF5, we established a RNF5 knockout cell line by utilizing the CRISPR-Cas9 system **(S4A Fig**). Consistent with previous studies [15], RNF5-deficiency promoted SeV-induced activation of the *IFNB* promoter and ISRE **(S4B Fig**). Importantly, we found that down-regulation of VISA as well as impaired phosphorylation of IRF3 caused by knockdown of iRhom2 was markedly rescued by RNF5-deficiency in un-infected and early-infected (4 h) but not late-infected (12 h) cells (**Fig 5I)**. Moreover, inhibition of SeV-triggered transcription of *IFNB* caused by knockdown of iRhom2 was also restored in RNF5-deficient cells (**Fig 5J)**. These results suggest that iRhom2 maintains VISA stability in un-infected and early-infected cells by down-regulating the level of RNF5.

### iRhom2 promotes the auto-polyubiquitination and degradation of RNF5

**Fig 6:**
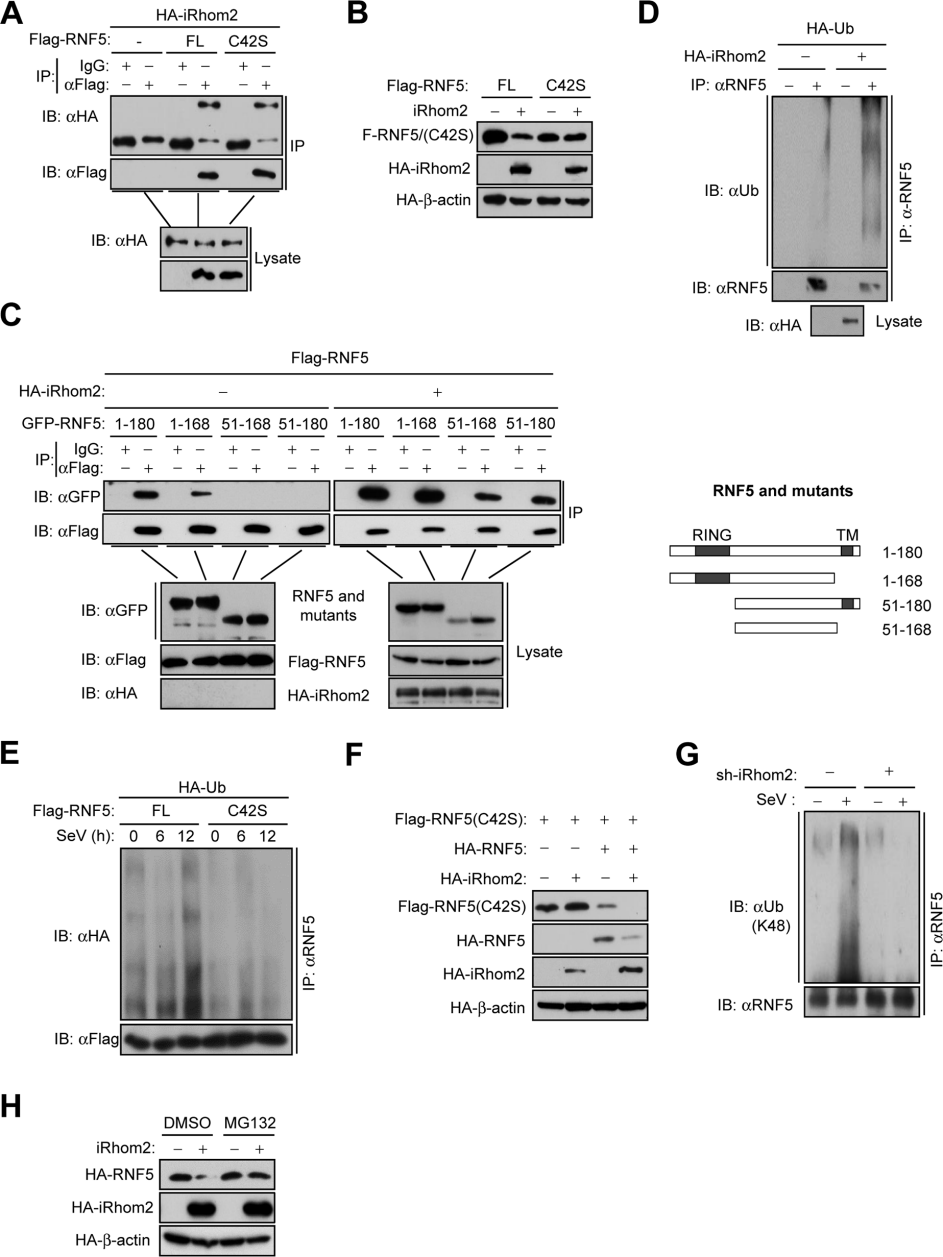
iRhom2 promotes the auto-ubiquitination and degradation of RNF5. (A) Co-immunoprecipitation and immunoblot analysis using the indicated antibodies with lysates of HEK293T cells transfected with the indicated plasmids. (B) Immunoblot analysis of the indicated proteins in HEK293T cells transfected with the indicated plasmids. (C) Co-immunoprecipitation and immunoblot analysis using the indicated antibodies with lysates of HEK293T cells transfected with the indicated plasmids. The schematic representations of RNF5 truncations are shown on the right. (D) Detection of RNF5 ubiquitination in HEK293T cells transfected with the indicated plasmids by co-immunoprecipitation and immunoblot analysis. (E) Detection of RNF5 ubiquitination by co-immunoprecipitation and immunoblot analysis with HEK293T cells transfected with the indicated plasmids and infected with SeV for the indicated times. (F) Immunoblot analysis of the indicated proteins in HEK293T cells transfected with the indicated plasmids. (G) Detection of endogenous K48-linked polyubiquitination of RNF5 by co-immunoprecipitation and immunoblot analysis with HEK293T cells transfected with controlled shRNA or iRhom2-shRNA and infected with SeV for 6 h. (H) Immunoblot analysis of the indicated proteins in HEK293T cells transfected with the indicated plasmids and then untreated or treated with MG132 (100 μM) for 6 h. FL, full length. Data are representative of three independent experiments with three biological replicates.

We next determined whether iRhom2 down-regulates the level of RNF5 by promoting its degradation. We found that iRhom2 interacted with RNF5 and such interaction was independent of the E3 ligase activity of RNF5, because the enzymatically inactive RNF5(C42S) mutant also associated with iRhom2 (**Fig 6A**). However, we noticed that the E3 ligase activity of RNF5 was required for its down-regulation by iRhom2 (**Fig 6B**). Therefore, we speculated that iRhom2 may promote the auto-polyubiquitination of RNF5, which subsequently results in its degradation. To test this hypothesis, we first examined the self-association of RNF5. The results showed that RNF5 could form homophilic complexes, which requires its N-terminal RING domain. Interestingly, we found that iRhom2 increased the self-association of RNF5 (**Fig 6C**), and promoted the polyubiquitination of endogenous RNF5 (**Fig 6D)**. Furthermore, mutation of C42 of RNF5 abolished its polyubiquitination before and after SeV infection (**Fig 6E)**. Interestingly, in co-transfection experiments, iRhom2 failed to down-regulate RNF(C42S), but could enhance wild type RNF5-mediated down-regulation of RNF5(C42S) **(Fig 6F**), indicating that iRhom2 promotes autoubiquitination and degradation of RNF5. Consistently, we found that K48-linked polyubiquitination of endogenous RNF5 after SeV infection was markedly impaired in iRhom2-knockdown cells compared with the control cells **(Fig 6G)**, and iRhom2-mediated down-regulation of RNF5 was inhibited in MG132 treated cells (**Fig 6H**). Collectively, these data suggest that iRhom2 promotes proteasomal degradation of RNF5 by mediating its self-association and K48-linked auto-polyubiquitination.

### ERAD is involved in the regulation of VISA

Recently, several studies have reported that ERAD is involved in regulation of innate immune responses [25,26]. RNF5 has been reported to act as a mammalian ERAD ligase that regulates the folding of cystic fibrosis transmembrane conductance regulator (CFTR) [27]. A more recent study demonstrates that the ERAD ligase GP78/AMFR regulates VISA-mediated signaling by unknown mechanisms [25]. These clues prompted us to investigate whether ERAD regulates the stability of VISA. Interestingly, the ERAD inhibitors DBeQ, NMS-873 and Eeyarestain [28–30] increased VISA-mediated activation of the *IFNB* promoter and ISRE **(S5A Fig**). Moreover, these ERAD inhibitors increased basal VISA levels and inhibited virus-induced VISA degradation at early phase of infection **(S5B Fig**). In addition, inhibition of the ERAD pathway potentiated the transcription of *IFNB*, *IP10* and *ISG56* genes before and after SeV infection **(S5C Fig**). These data suggest that inhibition of ERAD potentiates VISA stability and VISA-mediated signaling.

**Fig 7:**
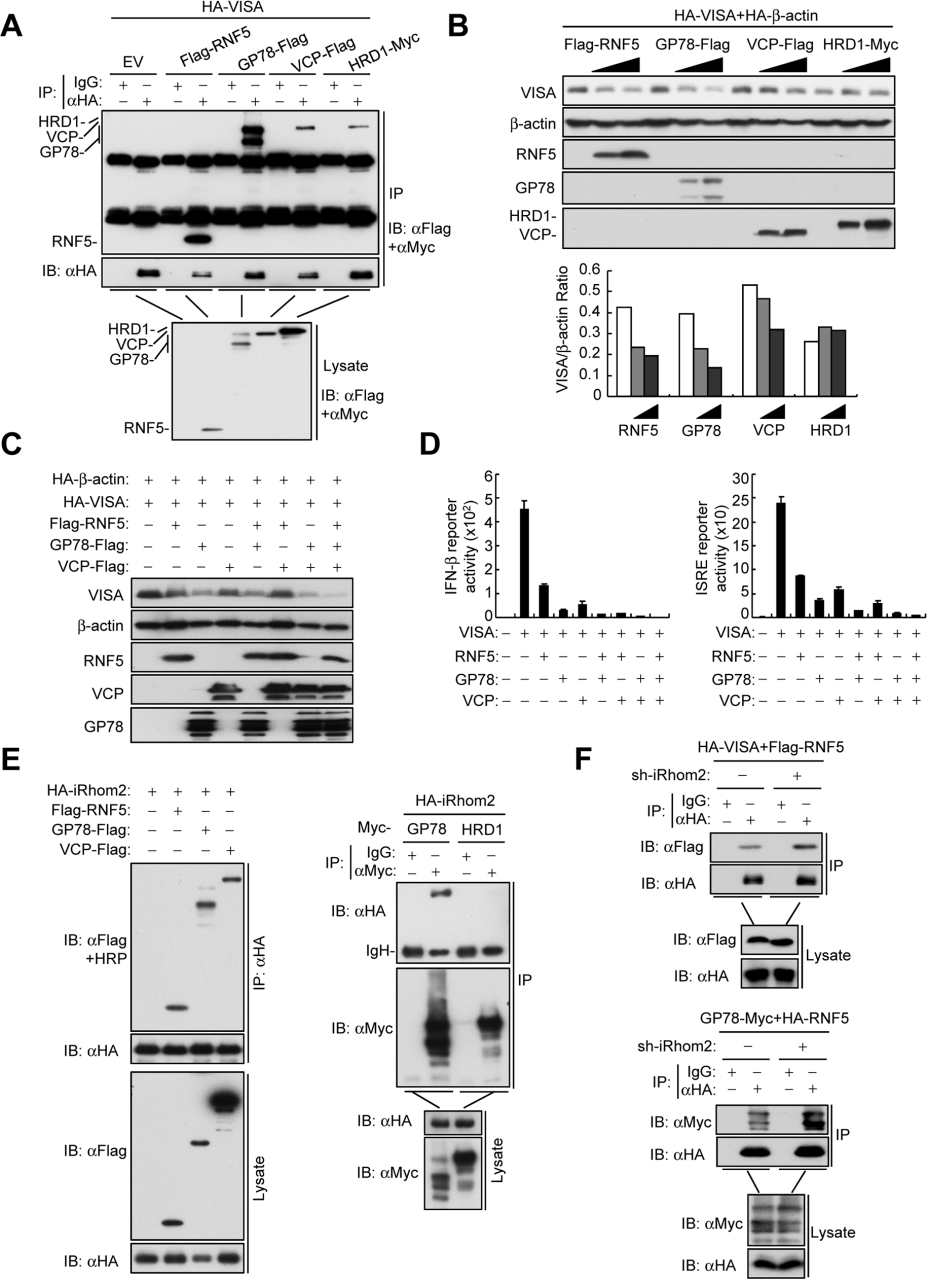
iRhom2 suppresses ERAD of VISA. (A) Co-immunoprecipitation and immunoblot analysis using the indicated antibodies with lysates of HEK293T cells transfected with the indicated plasmids. (B) Detection of effects of RNF5, GP78, VCP and HRD1 on the expression of VISA in HEK293T cells transfected with the indicated plasmids by immunoblot analysis with the indicated antibodies. Densitometry quantification was made with ImageJ Software (lower panel) (C) Immunoblot analysis of the indicated proteins in HEK293T cells transfected with the indicated plasmids. (D) IFN-β and ISRE reporter assays with HEK293T cells transfected with the indicated plasmids. **(E&F)** Co-immunoprecipitation and immunoblot analysis using the indicated antibodies with lysates of HEK293T cells transfected with the indicated plasmids.

Following these findings, we next determined whether VISA is associated with ERAD components including the mammalian ERAD ligases RNF5, GP78 and HRD1 and an ERAD-related AAA^+^ ATPase VCP (also known as p97/CDC48). The results showed that VISA was associated with RNF5, GP78, HRD1 and VCP (**Fig 7A)**. Moreover, endogenous co-immunoprecipitation experiments showed that the association of VISA and RNF5 were detected in non-infected and early-infected cells but decreased in late-infected cells (**S6A Fig**). Similar to RNF5, we found that GP78 and VCP but hot HRD1 down-regulated the levels of VISA in a dose-dependent manner (**Fig 7B)**. Conversely, knockout or knockdown of RNF5, GP78 and VCP enhanced the levels of endogenous VISA before and after SeV infection, and increased the levels of phosphorylated IRF3 and STAT1 (**S6B–S6D Fig)**. Moreover, GP78 and VCP cooperated with RNF5 in the down-regulation of VISA (**Fig 7C)**. Reporter assays indicated that RNF5, GP78 and VCP alone inhibited VISA-mediated activation of the *IFNB* promoter and ISRE and a combination of RNF5, GP78 and VCP abolished VISA-mediated signaling (**Fig 7D)**. These results suggest that the ERAD pathway, which consists of RNF5, GP78 and VCP, down-regulates VISA before and after viral infection.

Previously, it has been reported that GP78 has E4-like ubiquitin ligase activity that cooperates with RNF5 in the ERAD of CFTRΔF508 [31]. It is also well established that ubiquitinated ERAD substrates are dislocated from the membrane by VCP, which then hands substrates off to the proteasomes with the assistance of several cofactors [32]. We found that knockdown of GP78 inhibited RNF5-mediated K48-linked polyubiquitination of VISA (**S7A Fig)**. It has been demonstrated that RNF5 targets VISA at K362 and K461 for ubiquitination and degradation [15]. Our data showed that both VCP and GP78 down-regulated wild-type VISA but had little effects on its K362R, K461R and K362/K461R mutants (**S7B Fig)**. In addition, knockdown of GP78 but not RNF5 and iRhom2 inhibited the association of VCP and VISA (**S7C Fig)**. Taken together, these results suggest that GP78 and VCP act as downstream cofactors of RNF5 that promote K48-linked polyubiquitination of VISA and its degradation via the ERAD pathway.

### iRhom2 suppresses the ERAD of VISA

Recently, it has been reported that *Drosophila* iRhoms regulate the epidermal growth factor receptor pathway through ERAD[33]. These findings prompted us to test the hypothesis that iRhom2 maintains the stability of VISA by suppressing the ERAD pathway. Similar to VISA, we found that iRhom2 was also associated with RNF5, GP78 and VCP but not HRD1 (**Fig 7E**). Knockdown of iRhom2 enhanced the association of VISA with RNF5, but not with GP78 and VCP (**Fig 7F and S7D Fig)**. Knockdown of iRhom2 also increased the association of RNF5 with GP78 but not with VCP (**Fig 7F and S7D Fig**). However, knockdown of iRhom2 had no effects on the association of GP78 and VCP (**S7D Fig**). These results suggest that iRhom2 suppresses the association of VISA with the ERAD complexes.

### iRhom2 inhibits virus-triggered MAD of VISA

**Fig 8:**
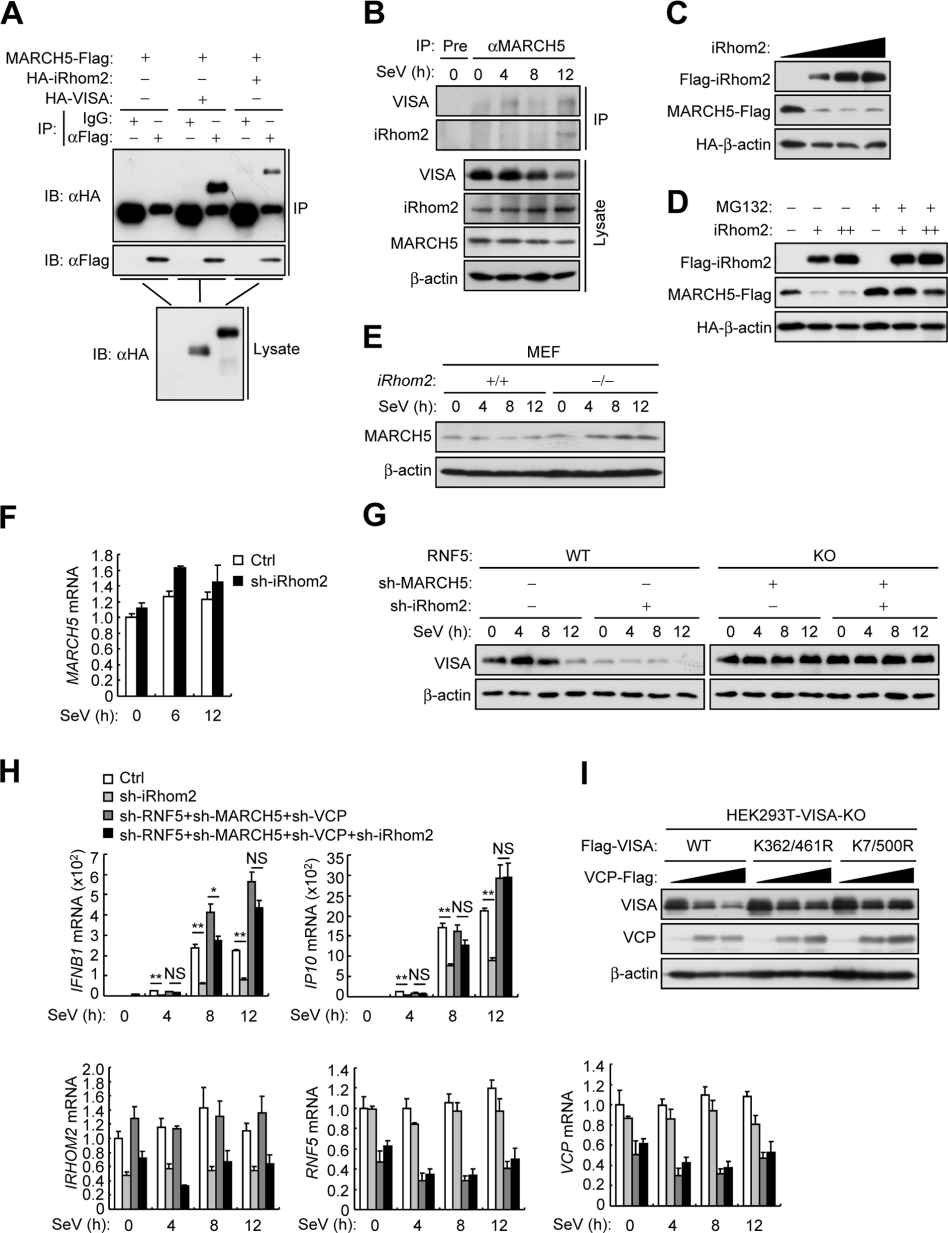
iRhom2 inhibits virus-triggered MAD of VISA. (A) Co-immunoprecipitation and immunoblot analysis using the indicated antibodies with lysates of HEK293T cells transfected with the indicated plasmids. (B) Co-immunoprecipitation and immunoblot analysis using the indicated antibodies with lysates of HEK293T cells infected with SeV for the indicated times. (C) Detection of dose-dependent effects of iRhom2 on the level of MARCH5 in HEK293T cells transfected with the indicated plasmids by immunoblot analysis with the indicated antibodies. (D) The experiments were similarly performed as in (C) except that cells were untreated or treated with MG132 (100 μM) for 6 h before immunoblot analysis. +: 0.5μg, ++: 1μg. (E) Immunoblot analysis of the indicated proteins in *iRhom2*^+/+^ and *iRhom2*^–/–^MEFs infected with SeV for the indicated times. (F) qPCR analysis of *MARCH5* mRNA in HEK293T cells transfected with iRhom2-shRNA, selected with puromycin (1 μg/ml) for 36 h and then infected with SeV for the indicated times. (G) Immunoblot analysis of endogenous VISA in wild-type and RNF5-KO HEK293T cells transfected with the indicated shRNA plasmids for 2 days and then infected with SeV for the indicated times. (H) qPCR analysis of *IFNB1* and *IP10* mRNAs in HEK293T cells transfected with the indicated shRNA plasmids, selected with puromycin (1 μg/ml) for 2 days and then infected with SeV for 5 h. (I) Immunoblot analysis of the indicated proteins in VISA-KO HEK293T cells transfected with the indicated plasmids for 24 h. *P < 0.05; **P < 0.01 (unpaired *t*-test). Data are representative of three experiments with similar results.

In our earlier experiments, we found that virus-triggered degradation of VISA was accelerated in iRhom2 knockdown or knockout cells at late phase of infection (**Fig 5A and 5B**). In addition, RNF5-deficiency only rescued VISA degradation caused by iRhom2 knockdown in un-infected and early-infected (4 h) cells but not late-infected (8–12 h) cells (**Fig 5I**). These results suggest that other mechanisms are responsible for VISA degradation at the late phase of infection. Recently, the mitochondrial ubiquitin ligase MARCH5 has been identified to promote proteasome-mediated degradation of VISA and reduce VISA aggregation during viral infection [17]. We found that MARCH5 interacted with iRhom2 and VISA in both overexpression and endogenous co-immunoprecipitation experiments (**Fig 8A and 8B**). As previously described, we confirmed that MARCH5 associated with VISA in a virus infection-dependent manner. Notably, similar to its interaction with VISA, MARCH5 weakly interacted with iRhom2 in un-infected and early-infected cells but such interaction was enhanced in late-infected cells (**Fig 8B**). Overexpression of iRhom2 markedly reduced the protein level of MARCH5, and the proteasome inhibitor MG132 reversed this effect (**Fig 8C and 8D**). In addition, iRhom2-deficiency markedly up-regulated the protein but not mRNA level of endogenous MARCH5 (**Fig 8E and 8F**), which suggests that iRhom2 promotes proteasome-dependent degradation of MARCH5. Unlike RNF5 and GP78, we noticed that knockdown of MARCH5 mainly inhibited virus-triggered down-regulation of VISA at the late phase of infection (10 h) (**S6D Fig**). As expected, down-regulation of VISA caused by iRhom2 knockdown was completely blocked by simultaneous knockdown of RNF5 and MARCH5 in un-, early- and late-infected cells (**Fig 8G**). Consistently, simultaneous knockdown of RNF5, MARCH5 and VCP significantly increased SeV-triggered transcription of *IFNB* and *IP10* and reversed the inhibitory effects of iRhom2 knockdown on SeV-triggered induction of the downstream genes (**Fig 8H**). These data suggest that iRhom2 maintains VISA stability by sequentially antagonizing RNF5- and MARCH5-dependent pathways.

Recent reports suggest that VCP plays a critical role in mitochondrial-associated degradation (MAD) of outer membrane proteins [32,34]. We also noticed that knockdown of VCP enhanced the stability of VISA both in un-infected and viral infected cells (**S6D Fig**). Moreover, overexpression of VCP down-regulated VISA but had little effects on VISA (K7/500R), in which the MARCH5 targeted lysine residues are mutated to arginines (**Fig 8I**), suggesting that VCP-mediated MAD of VISA required the ubiquitination of VISA by MARCH5. Collectively, these results suggest iRhom2 inhibits virus-triggered MAD of VISA by prompting proteasome-dependent degradation of MARCH5 at the late phase of infection.

## Discussion

VISA is an adaptor protein that plays critical roles in the signaling of RNA virus-induced production of type I IFNs and proinflammatory cytokines [5,6]. The protein level of VISA is delicately regulated during infection to ensure the optimal activation and timely termination of innate immune responses. Several host factors have been reported to down-regulate VISA via ubiquitination-related mechanisms [35], but how VISA stability is maintained to ensure efficient innate antiviral response is still enigmatic. In this study, we found that iRhom2 acts as a checkpoint for the ERAD/MAD of VISA, which dynamically maintains the stability of VISA and ensures proper innate immune response to RNA virus.

Our experiments suggest that iRhom2 plays a key role in efficient innate immune responses to RNA viruses. Firstly, overexpression of iRhom2 potentiated SeV-triggered induction of IFN-β and downstream ISGs whereas knockdown of iRhom2 had opposite effects. Second, induction of IFN-β and IL-6 was impaired in *iRhom2*^*‒/‒*^ MEFs, mouse BMDMs and BMDCs in response to SeV infection. Third, serum cytokines (IFNα, IFNβ, TNFα, IL6 and CCL5) induced by VSV infection were severely impaired in *iRhom2*^*‒/‒*^ in comparison to *iRhom2*^*+/+*^ mice. Deficiency of iRhom2 caused higher viral replication and lower serum levels of cytokines in mice following VSV infection. In addition, *iRhom2*^*‒/‒*^ mice exhibited greater infiltration of immune cells and damage in the lungs and higher mortality in comparison to *iRhom2*^*+/+*^ mice.

The role of iRhom2 in innate immune responses to RNA viruses is mediated by its physical and functional association with VISA. In addition to physical interaction with VISA, iRhom2 also modulates the stability of VISA. Protein levels of VISA in iRhom2-deficient cells were markedly reduced before and after virus infection, indicating that iRhom2 maintains the stability of VISA in both un-infected and infected cells. Previous studies have reported that several E3 ubiquitin ligases such as AIP4, Smurf1, RNF5, PSMA7, TRIM25, and MARCH5 can mediate K48-linked polyubiquitination of VISA and therefore promote its degradation [35]. Interestingly, we found that among these E3 ligases, iRhom2 specifically reduced the protein levels of RNF5 and MARCH5 in different phases of viral infection.

RNF5 is a transmembrane protein predominantly localized at the ER. It has been reported that RNF5 acts as an E3 ligase of the ERAD machinery, which ubiquitinates substrates and promote their degradation by ERAD. Several lines of evidence suggest that RNF5-mediated ERAD can down-regulate VISA in un-infected and early-infected cells. Firstly, the ERAD inhibitors DBeQ, NMS-873 and Eeyarestain increased basal VISA levels and inhibited virus-induced VISA degradation in the early phase of infection. Second, VISA was associated with ERAD components RNF5, GP78 and VCP. Notably, the association of endogenous RNF5 and VISA was detected in un- and early-infected cells but decreased in late-infected cells. Third, overexpression of RNF5, GP78 or VCP down-regulated the levels of VISA in a dose-dependent manner. Interestingly, GP78 and VCP down-regulated wild-type VISA but had little effects on its K362R, K461R and K362/K461R mutants in which the RNF5-targeted polyubiquitination sites are mutated, suggesting that RNF5-medaited polyubiquitination of VISA is required for its degradation by GP78 and VCP. Furthermore, RNF5, GP78 and VCP cooperated in the degradation of VISA. A combination of RNF5, GP78 and VCP abolished VISA-mediated signaling. In contrast, knockout of RNF5 and GP78 increased the levels of endogenous VISA in un- and early-infected cells. Taken together, these results suggest that VISA undergoes polyubiquitination and degradation via the RNF5-GP78-VCP axis of ERAD pathway in un- and early-infected cells.

MARCH5 is a mitochondria-resident protein that has been demonstrated to function in the MAD machinery. It has been reported that following RNA virus infection, MARCH5 polyubiquitinates activated VISA aggregates and promotes their degradation via the MAD pathway, therefore negatively regulates VISA-mediated signaling and prevents excessive immune responses [17]. We found that different from the association between RNF5 and VISA, which occurs mostly in un-infected and early-infected cells, MARCH5 was associated with VISA at late phase of viral infection. Consistently, knockdown of MARCH5 had little effects on the protein levels of VISA in un- and early-infected cells but markedly increased VISA level in the late phase of infection. These results suggest that MARCH5 promotes MAD of VISA at the late phase of viral infection.

MAD is analogous to the ERAD pathway in that they both require the VCP to dislodge ubiquitinated proteins from organelle membranes and escort their degradation by the proteasomes. Our results showed that overexpression of VCP down-regulated VISA but had little effect on VISA (K362/461R) or VISA (K7/500R), in which the RNF5 or MARCH5 targeted lysine residues were mutated to arginines. These data suggest that both RNF5-VCP-midated ERAD and MARCH5-VCP-mdiated MAD are involved in the degradation of VISA.

Several lines of evidence suggest that iRhom2 maintains VISA levels by antagonizing RNF5- and MARCH5-mediated degradation of VISA. Firstly, overexpression of iRhom2 promoted degradation of RNF5 and MARCH5 in a dose dependent manner whereas iRhom2-deficiency increased RNF5 and MARCH5 levels. Furthermore, iRhom2 mediated degradation of RNF5 and MARCH5 was abolished by the proteasome inhibitor MG132. Second, iRhom2 interacted with RNF5 and MARCH5 respectively in overexpression and endogenous co-immunoprecipitation experiments.

iRhom2 antagonizes RNF5- and MARCH5-mediated VISA degradation in a temporal manner. iRhom2 was associated with RNF5 in un-infected and early infected cells and such association was markedly impaired in late-infected cells. In contrast, iRhom2 weakly interacted with MARCH5 in un-infected and early-infected cells but their association was obviously enhanced in late-infected cells. Notably, the pattern of iRhom2-RNF5 and iRhom2-MARCH5 associations is similar to that of VISA-RNF5 and VISA-MARCH5 associations. In addition, down-regulation of VISA level by knockdown of iRhom2 was markedly rescued by RNF5-deficiency in un-infected and early-infected cells and by MARCH5-deficiency in late-infected cells respectively. Consistently, RNF5-deficiency or simultaneous knockdown of RNF5, MARCH5 and VCP markedly increased SeV-triggered induction of *IFNB* and the downstream ISGs and reversed the inhibitory effects of iRhom2-deficiency. Collectively, these data suggest that iRhom2 promotes degradation of RNF5 and MARCH5 at un/early-infected and late-infected cells respectively, therefore maintain the stability of VISA.

Although iRhom2 promotes VISA stability by distinct mechanisms before and after viral infection, it is observed that the protein level of VISA level is down-regulated after viral infection. It is possible that after activation of VISA-mediated signaling at the early phase of viral infection, the release of the antagonizing effects of iRhom2 on RNF5 and/or MARCH enables them to degrade VISA. It is also possible that other factors/mechanisms are responsible for the down-regulation of VISA after viral infection. Maintenance of VISA level to a certain degree in the late phase of viral infection may be important for the cells ready for another round of antiviral response.

The activation of VISA has been reported to be tightly and delicately regulated by various mechanisms to ensure appropriate immune responses. Such regulatory mechanisms include cell type specific regulation, spatial regulation, temoporal regulation, etc. Our study indicated that the E3 ligase RNF5 mediated ERAD of VISA mainly functions in uninfected and early-infected cells whereas the E3 ligase MARCH5 mediated MAD of VISA mainly plays its role in late-infected cells. Our study demonstrated that iRhom2 antagonizes RNF5 and MARCH5 at different stages of infection respectively, representing one of the spatiotemporal mechanisms of VISA regulation.

In summary, our study suggests a spatiotemporal model on the role of iRhom2 in regulation of innate immune responses to RNA viruses. In un-infected and early infected cells, iRhom2 mediates auto-ubiquitination and degradation of RNF5 and impairs ERAD of VISA, which promotes innate antiviral response. In the late phase of viral infection, iRhom2 mediates proteasome-dependent degradation of MARCH5 and impairs MAD of VISA, which maintains VISA at a certain level for next round of antiviral response. Therefore, iRhom2 acts as a vital checkpoint for the ERAD/MAD of VISA, which is important to maintain the protein homeostasis of VISA that ensures proper innate immune responses to RNA viruses.

## Materials and methods

### Mice

*iRhom2* gene-null founder mice on a C57BL/6 background were purchased from KOMP (KNOCKOUT MOUSE PROJECT) Repository, UC DAVIS. *iRhom2*^–/–^mice were bred in specific pathogen–free conditions. Six to eight-week-old female mice were used in the experiments and littermates were used as controls. Experiments were conducted without blinding, with age- and sex-matched mice.

### Preparations of MEFs, BMDMs, and BMDCs

MEFs were prepared from day 13.5 C57BL/6 mice embryos and cultured in Dulbecco’s modified Eagle’s medium (DMEM) supplemented with 10% fetal bovine serum. Bone marrow cells were isolated from tibia and femur. For preparation of BMDMs, the bone marrow cells were cultured in 10% M-CSF-containing conditional medium from L929 cells for 3–5 days. For preparation of BMDCs, the bone marrow cells were cultured in medium containing murine GM-CSF (50 ng/ml) and IL-4 (10 ng/ml) for 6–9 days.

### Isolation of mouse lung fibroblasts

Primary lung fibroblasts were isolated from about 4–6-week-old mice. Lungs were minced and digested in calcium and magnesium–free HBSS containing 10 mg/ml type II collagenase (Worthington) and 20 mg/ml DNase I (Sigma) for 3 h at 37°C with shaking. Cell suspensions were filtered through progressively smaller cell strainers (100 and 40 mm), centrifuged at 1500 rpm for 4 min, and plated in culture medium (1:1 [v/v] DMEM/Ham’s F-12 containing 10% FBS, 15 mM HEPES, 2 mM L-glutamine, 50 U/ml penicillin, and 50 mg/ml streptomycin). After 1 h, adherent fibroblasts were rinsed with HBSS and cultured in media.

### Reagents, antibodies, cells and viruses

Poly(I:C) (InvivoGen); Recombinant IFN-γ (R&D Systems); cycloheximide (CHX), MG132, DBeQ, NMS-873 (Sigma-Aldrich); GM-CSF (peproTech); lipofectamine 2000 (Invitrogen); polybrene (Millipore); SYBR (BIO-RAD); dual-specific luciferase assay kit (Promega); ELISA kit for murine IFN-α and IFN-β (PBL); ELISA kits for murine TNF, IL-6 and CCL5 (Biolegend) were purchased from the indicated manufacturers.

Anti-Flag (F3165) and anti-β-actin (A2228) were from Sigma-Aldrich. Anti-phospho-IκBα (Ser32/36) (5A5), anti-phospho-IRF3 (Ser396) (4D4G), anti-Myc (D84C12), were from Cell Signaling Technology. Anti-HA (16B12) was from Covance.Anti-GP78 (16675-1-AP) was from Protein Tech. Anti-TBK1 (ab109735), anti-phospho-TBK1 (Ser172) (ab109272) and anti-MARCH5 (ab77585) were from Abcam. Anti-GP78 (F-3), anti-IRF3 (FL-425), anti-p65 (C-20), anti-IRF1, anti-STAT1, and anti-phospho-STAT1 (Tyr701) were from Santa Cruz Biotechnology. Anti-K48-linked polyubiquitin (17–10408) was from Millipore. The secondary antibody goat anti-mouse (31430) or goat anti-rabbit (31460) IgG1 conjugated to HRP were from Pierce. Anti-VISA, anti-iRhom2 and anti-RNF5 polyclonal antibodies were previously described [6,15,21]. Anti-VCP was generated by immunizing mice. Human Embryonic Kidney 293 (HEK293, CRL-1573), and Henrietta Lacks (HeLa, CCL-2) cells were purchased from ATCC. HEK293T cells were originally provided by G. Johnson (National Jewish Health, Denver, CO). SeV, VSV (Indiana Strain), NDV were previously described [36].

### Constructs

IFN-β and ISRE luciferase reporter plasmids, as well as mammalian expression plasmids for HA-, GFP- or Flag-tagged VISA, iRhom2, RNF5 and their mutants, RIG-I, MDA5, TBK1, IKKε, IRF3, STING were previously described[36,37]. Flag-tagged VCP, GP78, Sumrf1, TRIM25, PSMA7 were constructed by standard molecular biology techniques. Flag-tagged AIP4 was originally provided by Dr. Z.F. Jiang (Peking University, China). Myc-tagged GP78 and HRD1 were provided by Dr. C. Wang (Shanghai Institutes for Biological Sciences, CAS, China).

### Generation of RNF5 and GP78 CRISPR knockout cells

HEK293T cells were transduced with plentiCRISPRv2-RNF5-sgRNA and plentiCRISPRv2-GP78-sgRNA virus for five days. Puromycin-resistant individual clones were selected and analyzed by western blotting to determine the efficiency of RNF5 and GP78 knockout (**S4 Fig**). RNF5-KO Subclone #2 and #3, and GP78-KO Subclone #1 was designated as HEK293T RNF5-KO and HEK293T GP78-KO respectively, and used for the experiments in this paper. The sequences of the guide RNA target sites are as follows. RNF5 gRNA: 5’- CCGCTCGCGATTTGGCCCTT-3’; GP78 gRNA: 5’-ACAGGGACAGGACTCGACCG-3’;

### Transfection and reporter assays

HEK293 cells were transfected by standard calcium phosphate precipitation method. HeLa cells and MEFs were transfected by lipofectamine 2000. To normalize for transfection efficiency, 0.01 μg of pRL-TK (Renilla luciferase) reporter plasmid was added to each transfection. Luciferase assays were performed using a dual-specific luciferase assay kit.

### RNA interference

Double-stranded oligonucleotides corresponding to the target sequences were cloned into the pSuper.Retro-RNAi plasmid (Oligoengine). The following sequences were targeted for *VCP* mRNA: 5′- GTAATCTCTTCGAGGTATA-3′; *GP78* mRNA: 5′- ACGCTCAGTTGAAATAACA -3′; *HRD1* mRNA: 5′- CCGCCATGCTGCAGATCAA -3′. iRhom2- and RNF5-shRNA plasmids were previously described[15,21].

### qPCR

Total RNA was isolated for qPCR analysis to measure mRNA abundance of the indicated genes. Data shown are the relative abundance of the indicated mRNA derived from mouse or human cells normalized to that of *Actb* or *GAPDH* respectively. Gene-specific primer sequences were as described [21,37] or reported in **S1 Table**.

### ELISA

MEFs, BMDMs and BMDCs were stimulated with viruses or transfected with nucleic acid analogs for the indicated times. The culture media were collected for measurement of TNF, IL-6, and IFN-β by ELISA.

### Co-immunoprecipitation, immunoblot analysis and Native PAGE

HEK293 cells, MEFs, BMDCs or BMDMs were lysed in l ml NP-40 lysis buffer (20 mM Tris-HCl (pH 7.4), 150 mM NaCl, 1 mM EDTA, 1% Nonidet P-40, 10 μg/ml aprotinin, 10 μg/ml leupeptin, and 1 mM phenylmethylsulfonyl fluoride). Co-immunoprecipitation, immunoblot analysis and Native PAGE were performed as previously described [37].

### Subcellular fractionation

The cell fractionation experiments were performed as previously described [15]. In brief, MEFs cells (1x10^7^) left uninfected or infected with SeV for various time points were washed with PBS and lysed by bouncing for 40 times in 2 ml homogenization buffer (10mMTris-HCl [pH 7.4], 2mMMgCl2, 10 mM KCl, and 250 mM Sucrose). The homogenate was centrifuged at 500 g for 10 min twice, and the pellet (P5) was saved as crude nuclei. The supernatant (S5) was centrifuged at 5,000 g for 10 min for crude mitochondria (P5K) precipitation. The supernatant (S5K) was further centrifuged at 50,000 g for 60 min for S50K and P50K generation. The fractions of P5K and P50K were lysed in lysis buffer (20 mM Tris-HCl [pH 7.4], 150 mM NaCl, 1 mM EDTA, and 1% NP-40, protease inhibitor cocktail) for 20 min; this was followed by immunoprecipitation or immunoblot analysis.

### Confocal microscopy

Confocal microscopy was performed as previously described [21]. Briefly, cells were fixed with 4% paraformaldehyde for 10 min at 25°C and then permeabilized and stained with indicated antibodies by standard protocols. The stained cells were observed with a ZEISS confocal microscope under a 60× oil objective.

### Viral infection in mice

Age- and sex-matched wild-type and *iRhom2*^–/–^mice were infected by intraperitoneal injection with VSV. The viability of the infected mice was monitored for 17 days. The mouse serum was collected at 6 h and 12 h after infection for measurement of cytokine production by ELISA.

### Lung histology

Lungs from control or virus-infected mice were dissected, fixed in 10% phosphate-buffered formalin, embedded into paraffin, sectioned, stained with hematoxylin and eosin solution, and examined by light microscopy for histological changes.

### Viral plaque assay

Tissues were weighed and then homogenized for 3s x3 in DMEM medium. After homogenization, the brain suspensions were centrifuged at 1,620 g for 30 min, and the supernatants were used for plaque assays on monolayers of Vero cells seeded in 24-well plates. The cells were infected by incubation for 1 hr at 37°C with serial dilutions of brain suspensions. After 1 hr infection, 2% methylcellulose was overlaid, and the plates were incubated for about 48 hr. The overlay was removed, and cells were fixed with 4% paraformaldehyde for 15 min and stained with 1% crystal violet for 30 min before plaque counting.

### Detection of ubiquitin-modified protein

The experiments were performed as previously described [36]. Briefly, cell lysates were immunoprecipitated with the indicated antibodies. The immunoprecipitates were re-extracted in lysis buffer containing 1% SDS and denatured by heating for 5 min. The supernatants were diluted with NP-40 lysis buffer until the concentration of SDS was decreased to 0.1%, followed by re-immunoprecipitation with the indicated antibodies. Ubiquitin-modified proteins were detected by immunoblots with ubiquitin antibodies.

### Statistical analysis

Unpaired Student’s *t*-test was used for statistical analysis with Microsoft Excel and GraphPad Prism Software. For the mouse survival study, Kaplan-Meier survival curves were generated and analyzed by Log-Rank test; *P* < 0.05 was considered significant. Fluorescent-imaging analysis was performed in a blinded fashion. Densitometry quantification was made with ImageJ Software.

### Ethics statement

Animal care and use protocol adhered to the National Rgulations for the Administration of Affairs Concerning Experimental Animals. Protocols and procedures for mice study were approved by the Institutional Review Board of Wuhan Institute of Virology (WIVA31201501).

## Supporting information

S1 Fig iRhom2-deficiency suppresses RNA-virus-triggered induction of downstream antiviral genes. **(A)** qPCR analysis of *Ifnb1*, *Isg56* and *Il6* mRNAs in *iRhom2*^*+/+*^ and *iRhom2*^*‒/‒*^ MLFs infected with SeV for the indicated times (horizontal axes).**(B&C)** qPCR analysis of *Ifnb1*, *Isg56* and *Il6* mRNAs in *iRhom2*^*+/+*^ and *iRhom2*^*‒/‒*^ MEFs (**b**) and BMDCs (**c**) infected with VSV or NDV for 6 h.*P < 0.05; **P < 0.01 (unpaired *t*-test). Data are representative of three experiments with similar results.
(TIF)

S2 Fig iRhom2 is colocalized with VISA, RNF5, VCP and MARCH5. **(A)** Immunofluorescent staining of iRhom2 (red) and VISA (green) in HeLa cells transfected with Flag-VISA and HA-iRhom2 for 24 h and then left un-infected or infected with SeV for 6 h. Scale bars represent 10 μm.**(B)** Cell fractionation analysis of *iRhom2*^−/−^ MEFs reconstituted with murine iRhom2 untreated or infected with SeV for the indicated time points. The cellular fractions were analyzed by immunoblotting with the indicated antibodies.
(TIF)

S3 Fig Amino acid 409–435 of iRhom2 is essential for SeV-triggered signaling. **(A)** Reporter assays of *IFNB1* promoter activity in HEK293 cells transfected with the indicated plasmids for 24 h and then infected with SeV for 12 h.**(B)** qPCR analysis of *IFNB1* in HEK293 cells transfected with the indicated plasmids for 24 h and then infected with SeV for 6 h.*P < 0.05 (unpaired *t*-test). Data are representative of three experiments with similar results.
(TIF)

S4 Fig CRISPR-mediated knockout of RNF5 and GP78 in HEK293T cells. **(A)** Immunoblot analysis for examination of the knockout efficiency in RNF5-KO HEK293T cells.**(B)** Reporter assays for *IFNB1* promoter and ISRE activity in wild-type and RNF5-KO HEK293 cells transfected with the indicated reporter plasmids.**(C)** qPCR analysis of *IFNB1*, *IP10* and *ISG56* mRNAs in wild-type and GP78-KO HEK293 cells transfected with the indicated reporter plasmids.*P < 0.05; **P < 0.01 (unpaired *t*-test). Data are representative of three experiments with similar results.
(TIF)

S5 Fig ERAD inhibitors potentiate VISA-mediated signaling. **(A)** Reporter assays for *IFNB1* promoter and ISRE activity in HEK293 cells transfected with the indicated plasmids and then treated with the indicated doses of DBeQ, NMS-873 and Eeyarestain.**(B)** Immunoblot analysis of endogenous VISA in HEK293T cells pre-treated with the indicated doses of DBeQ and NMS-873 for 3 h and then infected with SeV for the indicated times. Densitometry quantification was made with ImageJ Software (lower panel).**(C)** qPCR analysis of *IFNB1*, *IP10* and *ISG56* mRNAs in HEK293T cells pre-treated with the indicated doses of DBeQ for 3 h and then infected with SeV for 4 h.*P < 0.05 (unpaired *t*-test). Data are representative of three experiments with similar results.
(TIF)

S6 Fig Dynamic regulation of VISA degradation by ERAD/MAD. **(A)** Immunoblot analysis of the indicated antibodies for proteins co-immunoprecipitated with anti-RNF5 from lysates of HEK293T cells infected with SeV for the indicated times.**(B)** Immunoblot analysis of the indicated proteins in wild-type (WT) and RNF5-KO HEK293 cells infected with SeV for the indicated times.**(C)** Immunoblot analysis of the indicated proteins in wild-type (WT) and GP78-KO HEK293 cells infected with SeV for the indicated times.**(D)** Immunoblot analysis of the indicated proteins in HEK293 cells transfected with control shRNA, MARCH5-shRNA and VCP-shRNA plasmids and selected with puromycin (1 μg/ml) for 2 days, and then infected with SeV for the indicated times.Data are representative of three experiments with similar results.
(TIF)

S7 Fig RNF5-GP78-VCP axis is involved in the stability of VISA. **(A)** Co-immunoprecipitation and immunoblot analysis for detection of K48-linked polyubiquitination of VISA in HEK293T cells transfected with the indicated plasmids and then un-infected or infected with SeV for 6 h.**(B)** Co-immunoprecipitation and immunoblot analysis for the interaction of HA-VISA and VCP-Flag in HEK293T cells transfected with the indicated shRNA plasmids for 2 days.**(C)** Immunoblot analysis of the indicated proteins in VISA-KO HEK293T cells transfected with the indicated plasmids for 24 h.**(D)** Co-immunoprecipitation and immunoblot analysis of the indicated proteins in HEK293T cells transfected with the indicated plasmids for 24 h.Data are representative of three experiments with similar results.
(TIF)

S1 Table Listing of q-PCR primer sequences used in this study. (DOCX)
