# Supplementary figures and images for: iRhom2 is essential for innate immunity to RNA virus by antagonizing ER- and mitochondria-associated degradation of VISA
Source: PLoS Pathog. 2017 Nov 20;13(11):e1006693. doi: 10.1371/journal.ppat.1006693 (PMC5722342; doi:10.1371/journal.ppat.1006693)

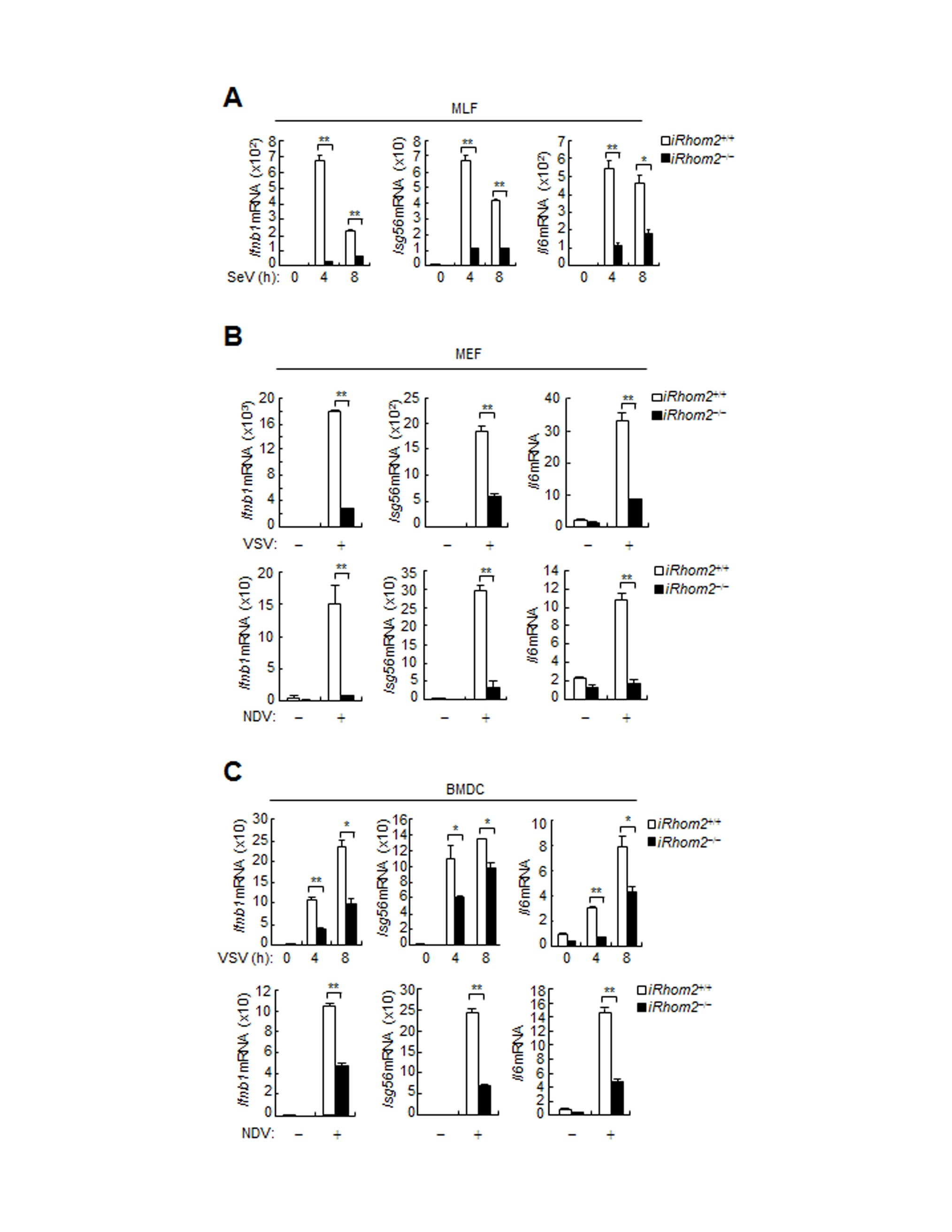

Supplement: S1 Fig — (A) qPCR analysis of Ifnb1, Isg56 and Il6 mRNAs in iRhom2+/+ and iRhom2‒/‒ MLFs infected with SeV for the indicated times (horizontal axes).(B&C) qPCR analysis of Ifnb1, Isg56 and Il6 mRNAs in iRhom2+/+ and iRhom2‒/‒ MEFs (b) and BMDCs (c) infected with VSV or NDV for 6 h.*P < 0.05; **P < 0.01 (unpaired t-test). Data are representative of three experiments with similar results. (TIF) [file ppat.1006693.s001.tif]

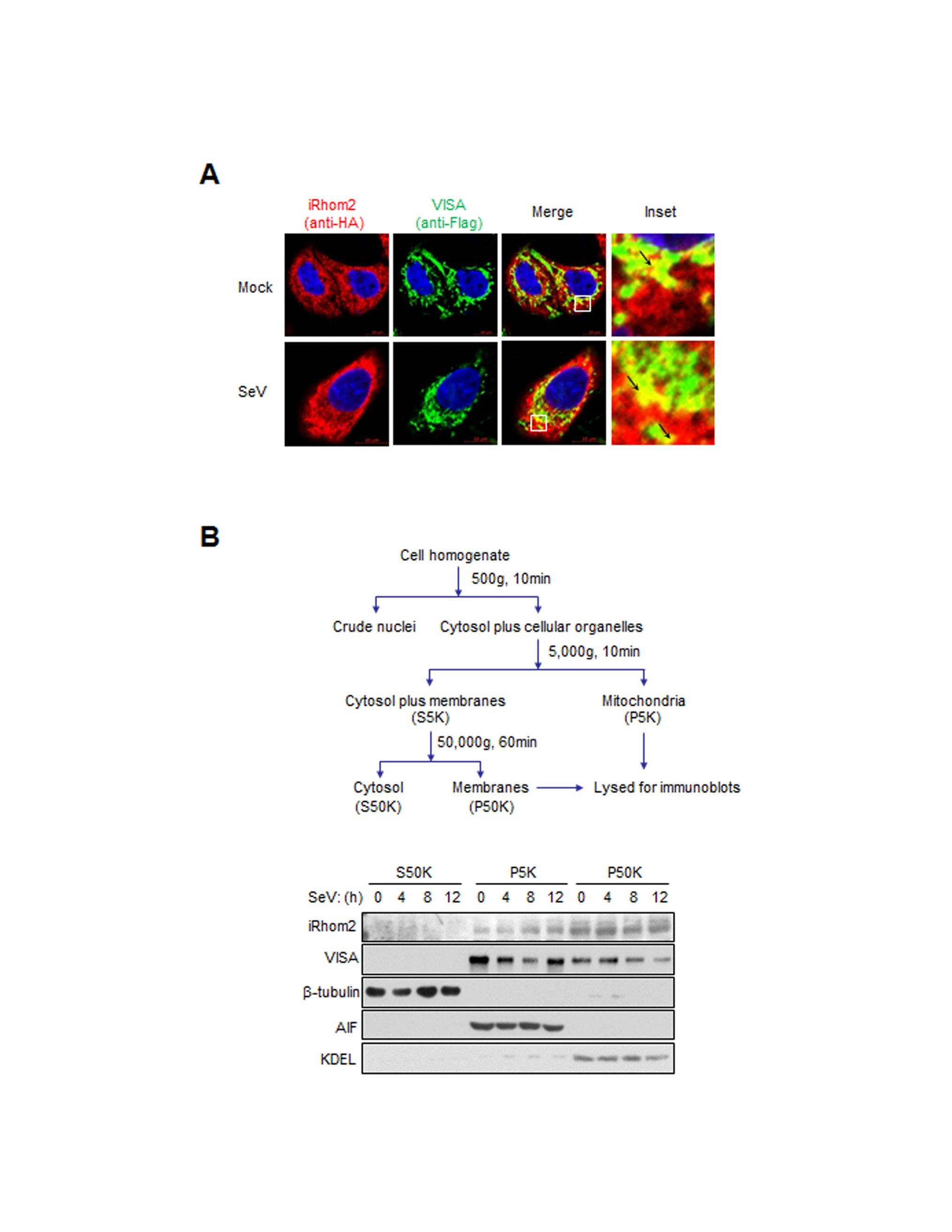

Supplement: S2 Fig — (A) Immunofluorescent staining of iRhom2 (red) and VISA (green) in HeLa cells transfected with Flag-VISA and HA-iRhom2 for 24 h and then left un-infected or infected with SeV for 6 h. Scale bars represent 10 μm.(B) Cell fractionation analysis of iRhom2−/− MEFs reconstituted with murine iRhom2 untreated or infected with SeV for the indicated time points. The cellular fractions were analyzed by immunoblotting with the indicated antibodies. (TIF) [file ppat.1006693.s002.tif]

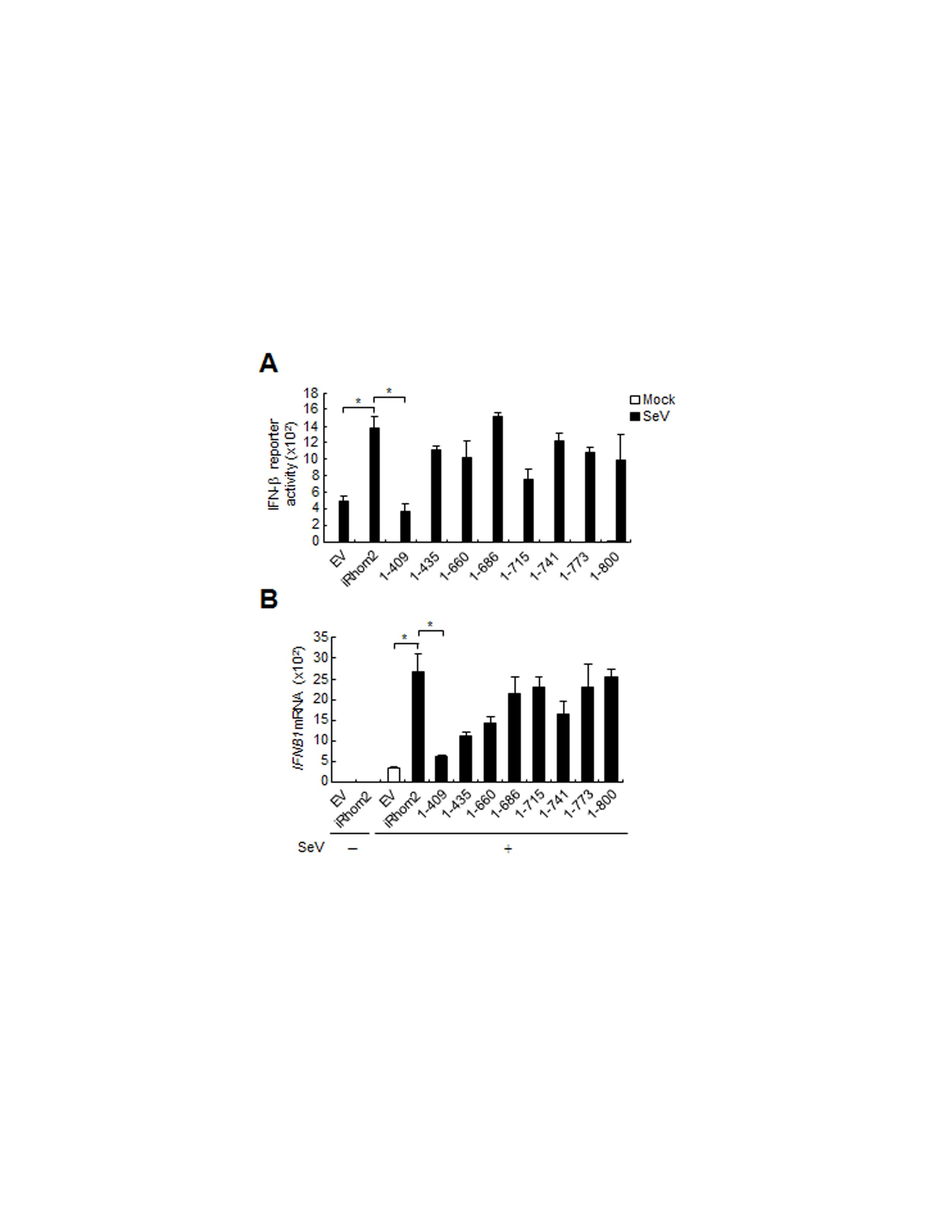

Supplement: S3 Fig — (A) Reporter assays of IFNB1 promoter activity in HEK293 cells transfected with the indicated plasmids for 24 h and then infected with SeV for 12 h.(B) qPCR analysis of IFNB1 in HEK293 cells transfected with the indicated plasmids for 24 h and then infected with SeV for 6 h.*P < 0.05 (unpaired t-test). Data are representative of three experiments with similar results. (TIF) [file ppat.1006693.s003.tif]

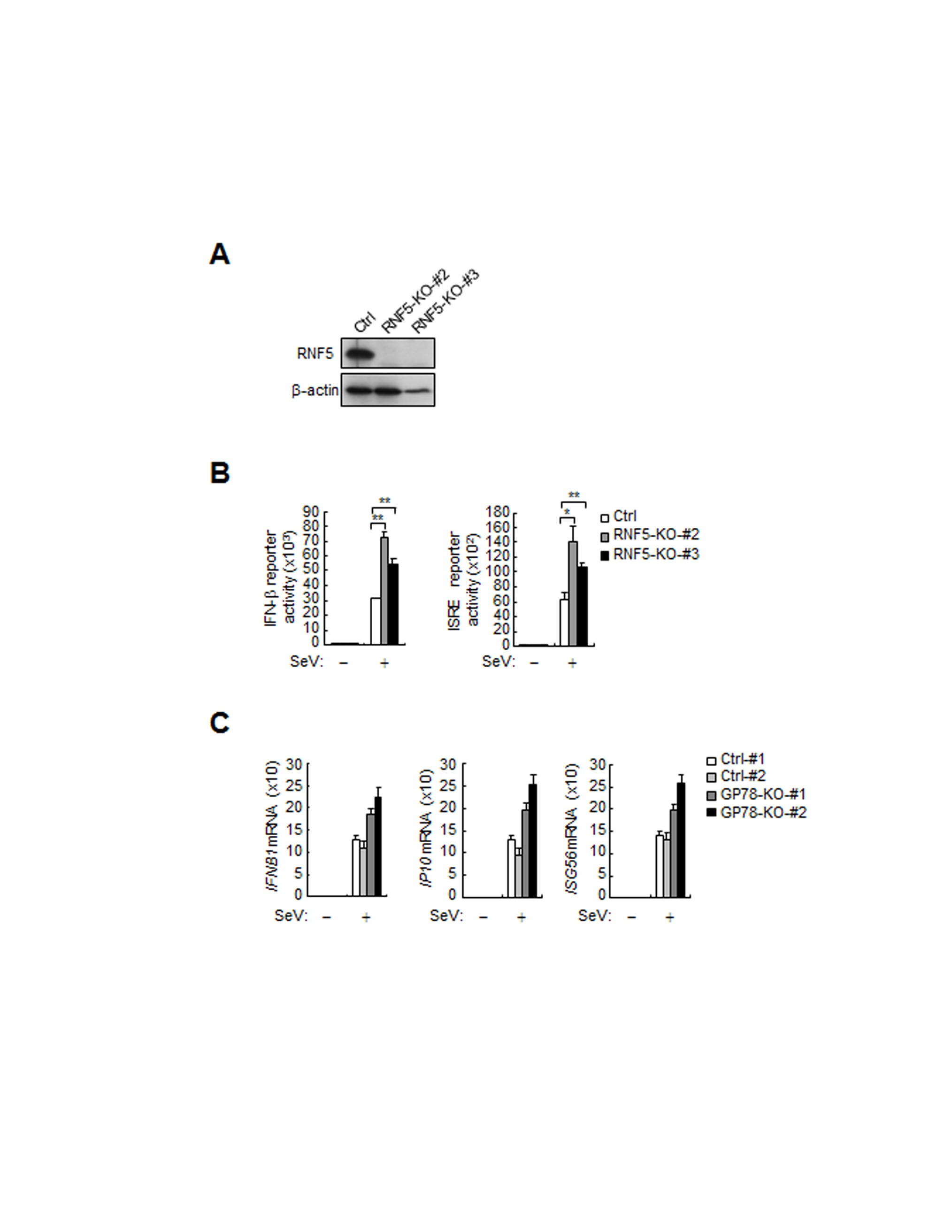

Supplement: S4 Fig — (A) Immunoblot analysis for examination of the knockout efficiency in RNF5-KO HEK293T cells.(B) Reporter assays for IFNB1 promoter and ISRE activity in wild-type and RNF5-KO HEK293 cells transfected with the indicated reporter plasmids.(C) qPCR analysis of IFNB1, IP10 and ISG56 mRNAs in wild-type and GP78-KO HEK293 cells transfected with the indicated reporter plasmids.*P < 0.05; **P < 0.01 (unpaired t-test). Data are representative of three experiments with similar results. (TIF) [file ppat.1006693.s004.tif]

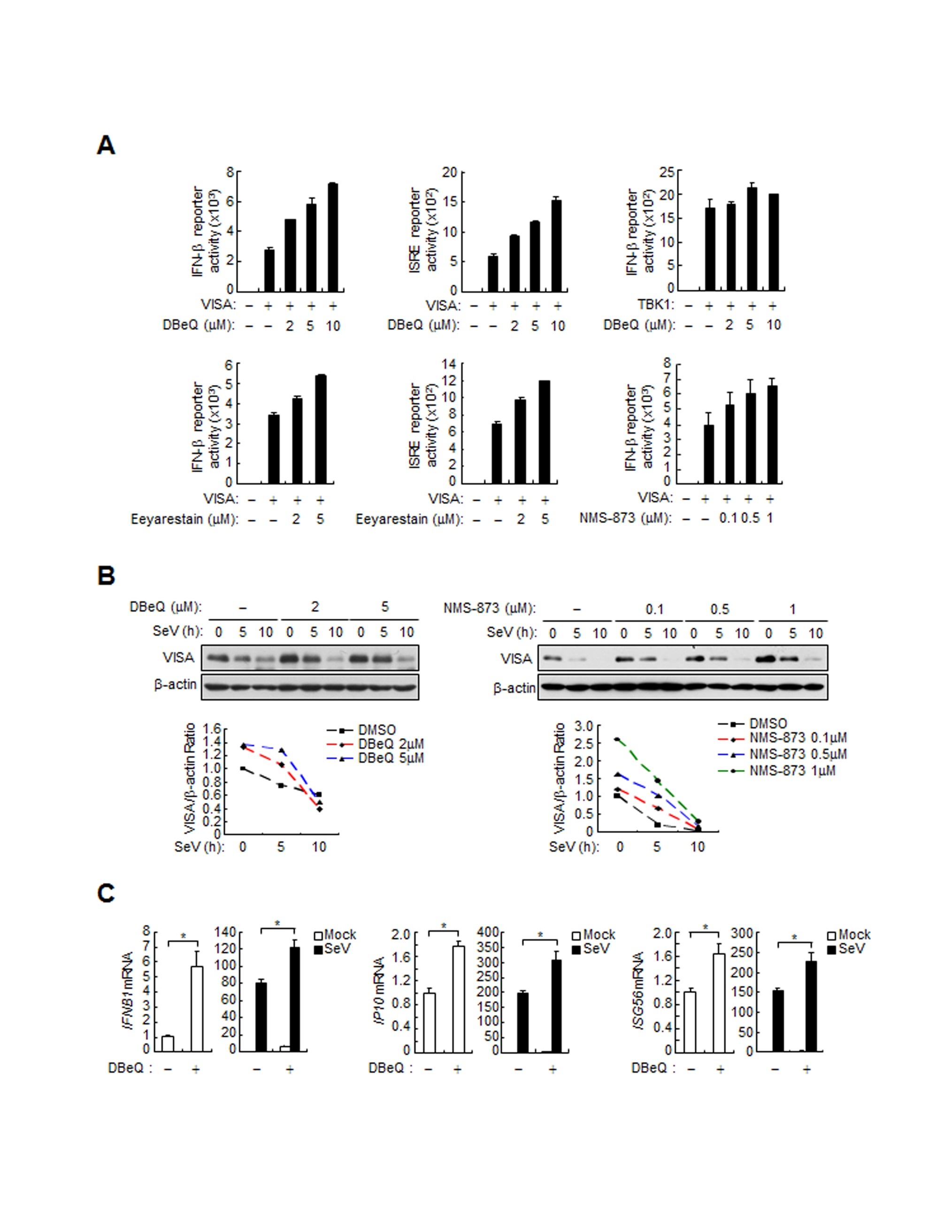

Supplement: S5 Fig — (A) Reporter assays for IFNB1 promoter and ISRE activity in HEK293 cells transfected with the indicated plasmids and then treated with the indicated doses of DBeQ, NMS-873 and Eeyarestain.(B) Immunoblot analysis of endogenous VISA in HEK293T cells pre-treated with the indicated doses of DBeQ and NMS-873 for 3 h and then infected with SeV for the indicated times. Densitometry quantification was made with ImageJ Software (lower panel).(C) qPCR analysis of IFNB1, IP10 and ISG56 mRNAs in HEK293T cells pre-treated with the indicated doses of DBeQ for 3 h and then infected with SeV for 4 h.*P < 0.05 (unpaired t-test). Data are representative of three experiments with similar results. (TIF) [file ppat.1006693.s005.tif]

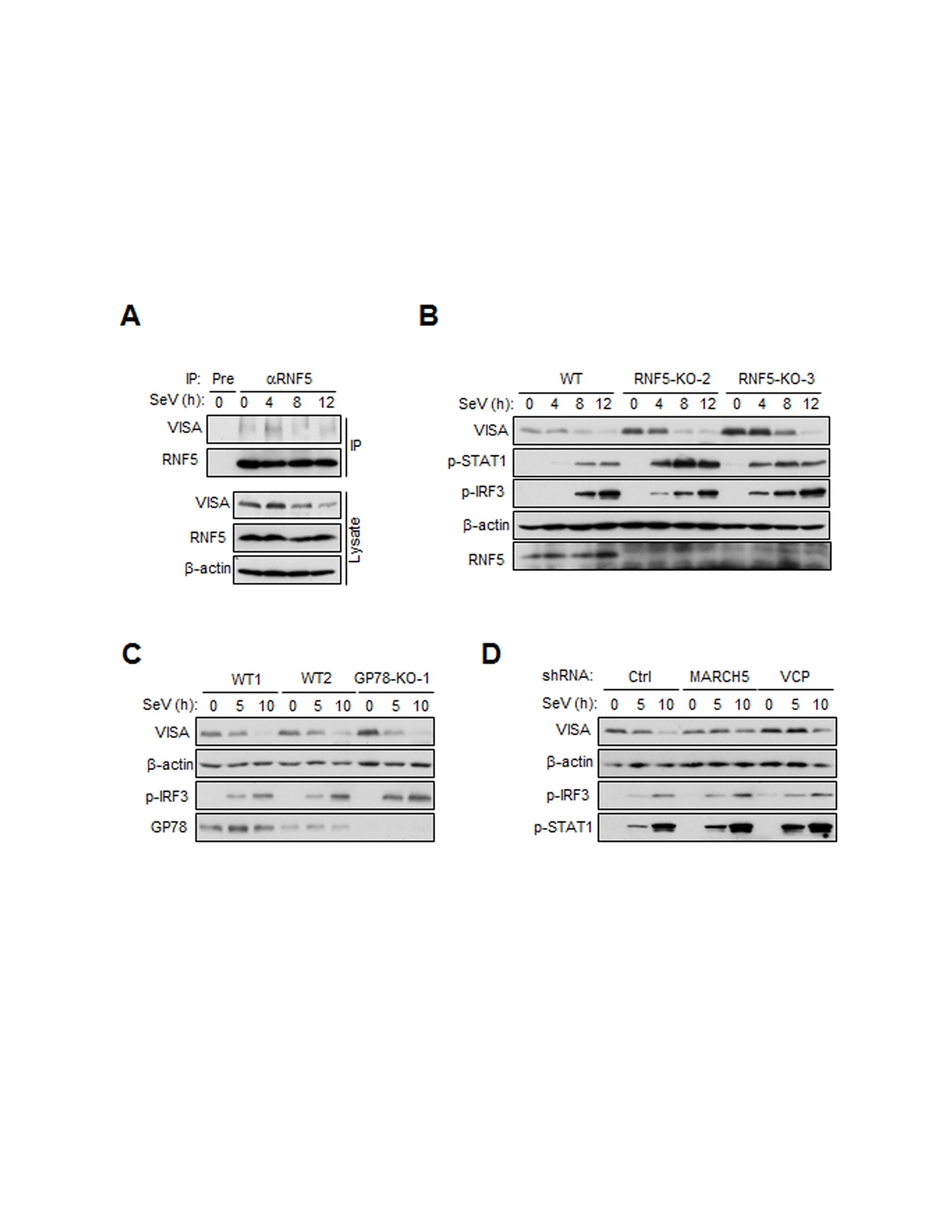

Supplement: S6 Fig — (A) Immunoblot analysis of the indicated antibodies for proteins co-immunoprecipitated with anti-RNF5 from lysates of HEK293T cells infected with SeV for the indicated times.(B) Immunoblot analysis of the indicated proteins in wild-type (WT) and RNF5-KO HEK293 cells infected with SeV for the indicated times.(C) Immunoblot analysis of the indicated proteins in wild-type (WT) and GP78-KO HEK293 cells infected with SeV for the indicated times.(D) Immunoblot analysis of the indicated proteins in HEK293 cells transfected with control shRNA, MARCH5-shRNA and VCP-shRNA plasmids and selected with puromycin (1 μg/ml) for 2 days, and then infected with SeV for the indicated times.Data are representative of three experiments with similar results. (TIF) [file ppat.1006693.s006.tif]

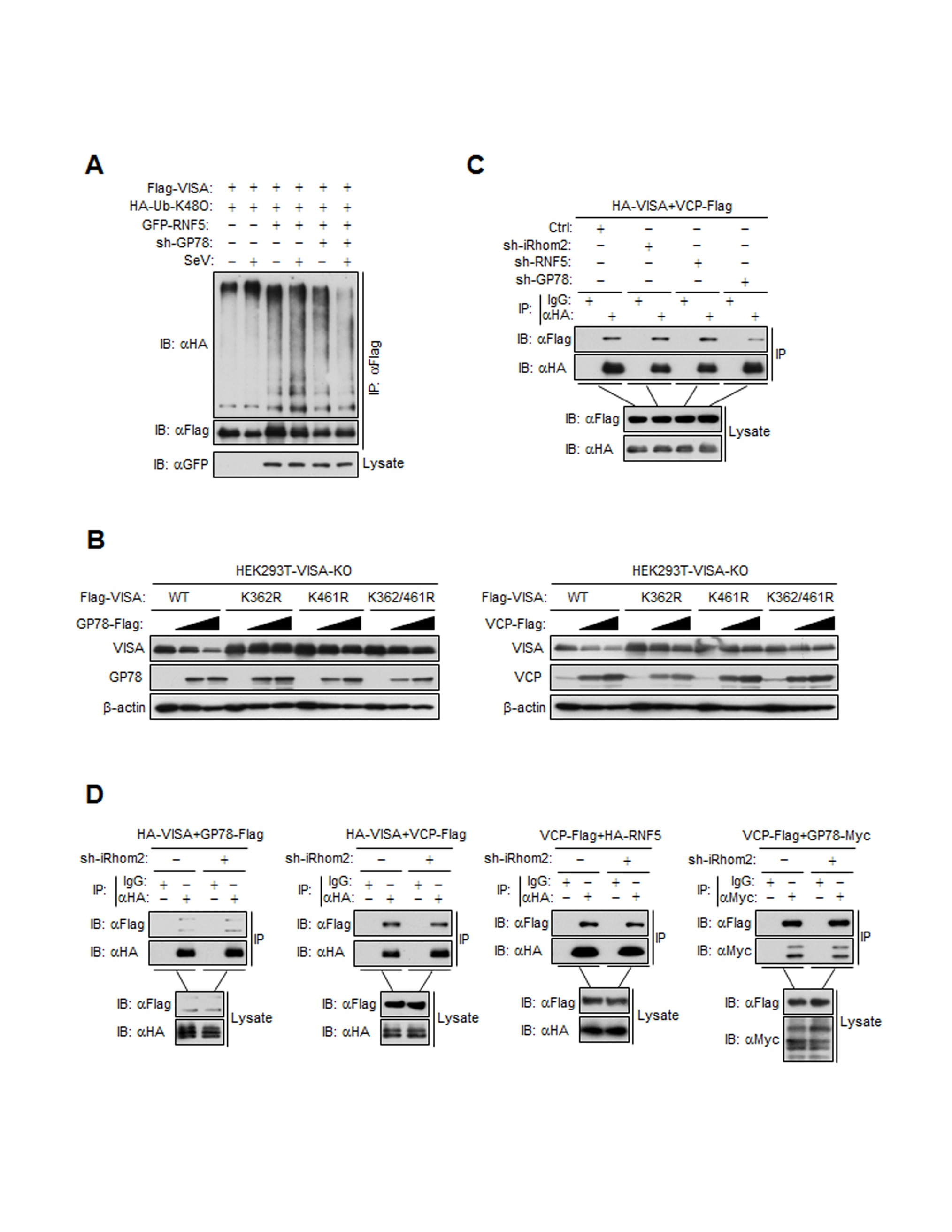

Supplement: S7 Fig — (A) Co-immunoprecipitation and immunoblot analysis for detection of K48-linked polyubiquitination of VISA in HEK293T cells transfected with the indicated plasmids and then un-infected or infected with SeV for 6 h.(B) Co-immunoprecipitation and immunoblot analysis for the interaction of HA-VISA and VCP-Flag in HEK293T cells transfected with the indicated shRNA plasmids for 2 days.(C) Immunoblot analysis of the indicated proteins in VISA-KO HEK293T cells transfected with the indicated plasmids for 24 h.(D) Co-immunoprecipitation and immunoblot analysis of the indicated proteins in HEK293T cells transfected with the indicated plasmids for 24 h.Data are representative of three experiments with similar results. (TIF) [file ppat.1006693.s007.tif]
