# Supplementary material for: iRhom2 is essential for innate immunity to RNA virus by antagonizing ER- and mitochondria-associated degradation of VISA
Source: PLoS Pathog. 2017 Nov 20;13(11):e1006693. doi: 10.1371/journal.ppat.1006693 (PMC5722342; doi:10.1371/journal.ppat.1006693)
Supplement: S1 Table — (DOCX) [file ppat.1006693.s008.docx]

| *Actb* Forward | CATTGCTGACAGGATGCAGAAGG |
| --- | --- |
| *Actb* Reverse | TGCTGGAAGGTGGACAGTGAGG |
| *GAPDH* Forward | GACAAGCTTCCCGTTCTCAG |
| *GAPDH* Reverse | GAGTCAACGGATTTGGTGGT |
| *Cxcl10* Forward | ATCATCCCTGCGAGCCTATCCT |
| *Cxcl10* Reverse | GACCTTTTTTGGCTAAACGCTTTC |
| *RHBDF2* Forward | CTACTGGCTGACCTTCGTCCAT |
| *RHBDF2* Reverse | ACTTCACGCTCTCGTACACACC |
| *IRF1* Forward | GAGGAGGTGAAAGACCAGAGCA |
| *IRF1* Reverse | TAGCATCTCGGCTGGACTTCGA |
| *GBP1* Forward | TAGCAGACTTCTGTTCCTACATCT |
| *GBP1* Reverse | CCACTGCTGATGGCATTGACGT |
| *Irf1* Forward | TCCAAGTCCAGCCGAGACACTA |
| *Irf1* Reverse | ACTGCTGTGGTCATCAGGTAGG |
| *Gbp2* Forward | AGATGCCCACAGAAACCCTCCA |
| *Gbp2* Reverse | AAGGCATCTCGCTTGGCTACCA |
| *RNF5* Forward | TGTTTGGAGACTGCTCGGGAAG |
| *RNF5* Reverse | ACTGGACACTCTTGCCGTTCTG |
| *MARCH5* Forward | AGCCGTGACTTATGGAGCAGTG |
| *MARCH5* Reverse | TATCAGCATGACAGGAATAGTAGG |
| *VCP* Forward | CTGGCAGATGATGTGGACTTGG |
| *VCP* Reverse | CAGAGCAGCCTCTGAACATAGG |
| *VSV-N* Forward | ACGGCGTACTTCCAGATGG |
| *VSV-N* Reverse | CTCGGTTCA AGATCCAGGT |

**Supplemental Table 1. qPCR Primers**
